# Asymmetries in the Acceptability and Felicity of English Negative Dependencies: Where Negative Concord and Negative Polarity (Do Not) Overlap

**DOI:** 10.3389/fpsyg.2019.02486

**Published:** 2019-11-12

**Authors:** Frances Blanchette, Cynthia Lukyanenko

**Affiliations:** ^1^Center for Language Science and Department of Psychology, Penn State University (PSU), University Park, TX, United States; ^2^Linguistics Program, Department of English, George Mason University, Fairfax, VA, United States

**Keywords:** acceptability, conditionals, experimental approaches, felicity, grammaticality, Negative Concord, Negative Polarity

## Abstract

Negative Concord (NC) constructions such as *the news anchor didn’t warn nobody about the floods* (meaning “the news anchor warned nobody”), in which two syntactic negations contribute a single semantic one, are stigmatized in English, while their Negative Polarity Item (NPI) variants, such as *the news anchor didn’t warn anybody about the floods*, are prescriptively correct. Because acceptability is often equated with grammaticality, this pattern has led linguists to treat NC as ungrammatical in “Standard” or standardized English (SE). However, it is possible that SE grammars do generate NC sentences, and their low incidence and acceptability is instead due to social factors. To explore this question, and the relationship between NC and NPI constructions, we compared the acceptability of overtly negative noun phrases (e.g., *nobody*), NPIs (e.g., *anybody)*, and bare plurals (e.g., *people*), in negative contexts and in conditionals. Negative items were followed by a consequence which supported their single negative meaning, while conditional items were followed by a consequence compatible with the NPI and the bare plural but not the negative noun phrase. Acceptability ratings of the critical NC sentences were reliably lower than constructions with NPIs and bare plurals, but the consequences for all three of these sentence types were rated highly. This reflects an asymmetry in participants’ acceptance of NC and their readiness to interpret it in context. A follow-up study with only conditionals revealed that speakers can also find NPIs infelicitous in conditional contexts with consequences that are compatible with a negative interpretation of the NPI, and that negative arguments are felicitous in these same contexts. Taken together, the results support the hypothesis that speakers who do not accept NC have grammars that generate both NC and NPI constructions, and further, that these speakers have two underlying structures for *any*-NPIs in English.

## Introduction

Human languages display diversity in whether and how they instantiate negative dependencies (Auwera and Alsenoy, 2016). In a subset of languages, negative arguments are typically found in Negative Concord (NC) constructions, in which two or more syntactic negations contribute a single semantic negation, as in the following Italian example from Zanuttini (1997, p. 8, ex. (13a)):


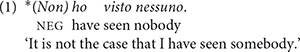


In (1), the preverbal negative marker *non* and the negative direct object argument *nessuno* “nobody” are interpreted as a single semantic negation, reflecting a pattern typical to NC constructions^1^.

Other languages instantiate negative dependencies through Negative Polarity Item (NPI) constructions^2^. These NPI constructions are similar to the NC construction seen in (1), but they do not have an overtly negative noun phrase. Instead, in place of a phrase like *nessuno* “nobody” in (1), they contain a phrase which is not overtly negative but depends on a preceding element, prototypically a negation, for its licensing. The following is example is from Ewe (Collins et al., 2017, p. 2, ex. (2b)):


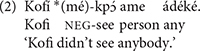


In (2), the term *ádéké* “any” is an NPI. It is not overtly marked for negation, but the negative marker *mé* is required for acceptability, in a manner similar to NC in Italian and other languages.

NC constructions are often modeled as a syntactic dependency between negative elements within a clause (e.g., Haegeman and Zanuttini, 1996; Zeijlstra, 2004; Déprez, 2011; Puskás, 2012; Blanchette, 2013). This is due to the requirement that the preverbal marker be present in the structure as in (1), in conjunction with the resumptive morphological marking of negation^3^. The grammatical nature of NPI constructions is subject to debate, but since Ladusaw (1979) a common view is that they primarily reflect a semantic-pragmatic dependency between the NPI and its licensing context [the negation in (2); e.g., Krifka, 1995; Giannakidou, 1998, 1999, 2002; Zwarts, 1998; Gajewski, 2011; Chierchia, 2013].

English is among the languages which instantiate both NC and NPI constructions. In vernacular English varieties, spontaneous speech reflects variation in negative contexts between these two structure types, as in the following examples from Tortora et al. (2017) *The Audio-Aligned and Parsed Corpus of Appalachian English* (AAPCAppE)^4^. (See Childs, 2017 for an analysis of this type of variation in British vernacular speech corpora.)


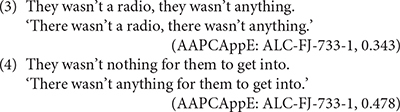


Speakers may even employ both construction types within a single utterance, as in the following example from an Appalachian English speaker (cited in Blanchette, 2016, p. 110):


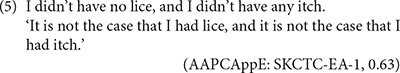


An important and distinguishing feature of English NC is its heavy social stigma (Horn, 2010), a stigma which is not present in other languages with NC. NC is often condemned as illogical, and “Standard” or standardized English (SE) speakers tend to avoid it in usage. Many linguists have taken its unacceptability and absence from SE usage to reflect its underlying ungrammaticality^5^. This is at least in part due to the traditional causal link assumed by linguists between acceptability and grammaticality on the one hand, and unacceptability and ungrammaticality on the other (Etxeberria et al., 2018, p. 2). If there exists a direct connection between acceptability and grammaticality, then it follows that SE grammars generate (prescriptively correct) NPI constructions, but they do not generate NC. Following this line of reasoning further leads to a hypothesis in which utterances such as (5) reflect a form of code-switching between two different grammatical systems. The Appalachian speaker controls two systems, and the component of her grammar that generates the NPI construction overlaps with SE grammars, while the component of her grammar that generates NC does not.

This paper uses experimental means to explore an alternative hypothesis, one which does not assume a direct and causal link between NC unacceptability and ungrammaticality (Lewis and Phillips, 2015; Etxeberria et al., 2018). We acknowledge the social forces shaping NC acceptability, and use measures of meaning in context to contribute toward our understanding of its grammaticality in relation to NPI constructions. We exploit the fact that NPI constructions appear in a broader range of contexts than NC, to illustrate how speakers who do not accept NC nevertheless demonstrate knowledge of when these constructions do and do not overlap in meaning with NPI constructions. We discuss how the results can be taken to support a theory of grammar in which utterances as in (5) do not reflect code-switching, but rather, a form of shifting between surface forms which reflect similar underlying grammatical mechanisms.

## English Negative Concord and Negative Polarity

This section summarizes several relevant grammatical theories and experimental and psycholinguistic studies of NC and NPI constructions. The literature is vast, and we focus on those most relevant to our experiments. We begin with the assumption that grammars are “abstract descriptions of the representations built by the cognitive system” during language comprehension and production, rather than cognitively real, static references queried by the parser (Lewis and Phillips, 2015, p. 30). Social forces such as prescriptive pressure are external to cognitive representations, but they interact in crucial ways with the outputs of those representations. This is most relevant to studies of NC, which we summarize first.

### Negative Concord

#### The Syntactic Agree Approach

Many recent theories of NC model it as a syntactic Agree relation between negative elements within a clause (e.g., Zeijlstra, 2004; Puskás, 2012; Wallage, 2012; Espinal and Tubau, 2016; Tubau, 2016). Such theories are often motivated, at least in part, by the contrast between NC and so-called Double Negation (DN) constructions, in which each of two syntactic negations contributes a semantic negation. The following examples illustrate:


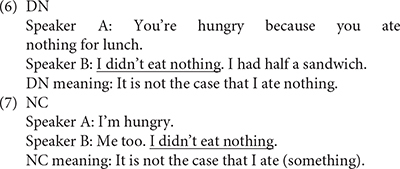


Zeijlstra (2004) proposes that sentences such as those in (6) and (7) instantiate two different grammatical systems. Déprez (2011) proposes instead that the distinction is more of a “micro-parametric” one, in which grammars may generate either NC or DN, depending on the syntactic configuration. This “micro-parametric” view is supported by recent experimental work, which has shown that in English as well as in Romance languages, DN constructions as in (6) exist alongside NC constructions as in (7), with DN being reliably associated with a marked prosodic tune relative to the single negation interpretation of NC (Espinal and Prieto, 2011; Espinal et al., 2016; Blanchette et al., 2018; Blanchette and Nadeu, 2018; Déprez and Yeaton, 2018).

Syntactic Agree approaches to modeling NC posit that negative elements are lexically endowed with an uninterpretable feature which needs to be checked in the syntax. Under an Agree approach, the NC sentence in (6) would be modeled roughly as follows (cf. Zeijlstra, 2004):


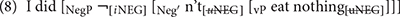


Example (8) shows how the negative noun phrase *nothing* and the marker *n’t* enter the structure with an uninterpretable negative feature [*u*NEG]. By virtue of being uninterpretable, these features must check themselves against the interpretable negative feature [*i*NEG] residing on a phonologically null operator in the head of a higher negative phrase (NegP). This checking relation establishes a syntactic dependency between the semantically non-negative elements *n’t* and *nothing* and the semantically negative null operator, yielding an NC structure with a single negative interpretation.

Tubau (2016) represents a recent Agree approach to modeling English NC. She notes that in British English dialects, negative noun phrases need not always be preceded by another negation, and shows how the following variant types are attested:

(9) I didn–t eat nothing. (NC)
(10) I ate nothing.

To explain the variation seen in (9) and (10), Tubau proposes a theory in which negative noun phrases such as *nothing* have two distinct lexical entries. The *nothing* in (9) is endowed with an uninterpretable [*u*NEG] feature, which triggers the concord (Agree) relation (as in (8) above), while the *nothing* in (10) has an interpretable [*i*NEG] feature, and thus contributes its own semantic negation without needing to establish an Agree relation with a preceding negative operator. Vernacular British English dialects differ from SE in this theory. In general, SE is assumed to be a DN language, having neither [*i*NEG] nor [*u*NEG]. Instead, each syntactic negation is taken to instantiate an underlying negative operator which is not featurally active and therefore never eligible for Agree, meaning that structures like (9) are not generated.

#### Negative Concord in Standardized (“Standard”) English

While vernacular English varieties are known for instantiating NC (Wolfram and Fasold, 1974; Nevalainen, 2006), a series of recent experimental studies show that SE speakers also have reliable intuitions about this construction type. The studies show that SE speakers have a clear knowledge of the syntactic distribution of NC (Blanchette, 2017), an understanding of its meaning and prosodic properties in relation to DN (Blanchette et al., 2018), and an apparent proclivity toward building NC structures during online processing (Blanchette and Lukyanenko, 2019). These studies all involve comparison of sentences with a negative noun phrase in direct object position following a negative marker as in (11) (and (6/7) above), and sentences with a negative noun phrase in canonical subject position preceding a negative marker, as in (12)^6^ ^,7^ :

(11) I didn’t see nobody.
(12) Nobody didn’t see me.

The results of all three studies demonstrate that SE speakers reliably prefer NC interpretations for sentences like (11), but DN interpretations for sentences like (12).

The sentences in (11) and (12) illustrate a typological divide between what Giannakidou (1998) categorizes as “non-strict” and “strict” NC. Both strict and non-strict NC languages have sentences like (11), in which a negative noun phrase is preceded by and acts in concord with a negated auxiliary, but only strict NC languages have sentences like (12), in which the negative noun phrase both precedes and acts in concord with the negated auxiliary. On the basis of their findings, Blanchette and Lukyanenko (2019, p. 24) therefore suggest that SE may be categorized as “non-strict^8^.” They further note a similarity between speakers’ subtle intuitions about NC in SE, and more obvious intuitions about parallel NPI constructions. To illustrate, consider the following contrast:

(13) I didn’t see anybody.
(14) *Anybody didn’t see me^9^.

Example (14) shows that NPIs are unacceptable in canonical subject position. Note that (13), which is acceptable, is equivalent in meaning and nearly identical in form to (11), while unacceptable (14) is nearly identical in form to (12)^10^. The acceptability of NPI constructions thus parallels speakers’ intuitions about NC, suggesting a possible grammatical relationship between these two construction types. The studies we report in this paper take a first step toward understanding the nature of this relationship, and how it might inform abstract grammatical as well as cognitive theories. To illustrate this, we next provide some background on NPI constructions.

### Negative Polarity

#### Downward Entailingness

Ladusaw (1979) observed that NPIs are acceptable when they occur in the scope of a downward entailing expression, which creates “a semantic context which makes inferences run downward on a scale” (p. 179)^11^. The following examples illustrate that negation is downward entailing:

(15) Maria didn’t drive.
(16) Maria didn’t drive fast.
(17) Maria didn’t drive fast and furiously.

The sets denoted by the predicate narrow from (15) to (17), and the entailments hold in that downward direction: If Maria did not drive (the widest set), then it must also be true that she did not drive fast (a narrower set), and that she did not drive fast and furiously (the narrowest set). Note that removing the negation voids this entailment pattern:

(18) Maria drove.
(19) Maria drove fast.
(20) Maria drove fast and furiously.

It can be true that Maria drove, but that she drove slowly and cautiously, which means that (18) being true does not entail that (19) and (20) are also true.

Negation’s ability to trigger downward entailing inferences, Ladusaw proposes, is the property that allows it to license NPIs, its removal leading to unacceptability:

(21) Mary didn’t drive any cars.
(22) *Mary drove any cars^12^.

In addition to this semantic specification, there is also thought to be a syntactic requirement that the NPI be c-commanded by its licensor (Baker, 1970, as cited in Linebarger, 1987, p. 330). Sentence (14), in which the NPI precedes the negation, is one example of why the c-command requirement is needed, since an eligible licensor is present in the structure, but the sentence is nevertheless unacceptable.

Further research on downward entailment for NPI licensing has revealed a number of apparent exceptions to the pattern, one of which is conditionals, which we employ in our experiment. The lack of straightforward downward entailingness in these contexts has led semanticists to expand, refine, or propose alternatives to this as a licensing condition (Giannakidou, 1998, 1999; Von Fintel, 1999; Gajewski, 2011; Chierchia, 2013).

Some recent psycholinguistic studies of NPIs have assumed the downward entailingness theory of NPI licensing in examining speakers’ processing of NPIs. Both Vasishth et al. (2008) and Parker and Phillips (2016), for example, investigate so-called “NPI illusions” in which speakers accept and successfully process NPIs despite their not being in the c-command domain of a preceding downward entailing licensor. Interestingly, however, when Szabolsci et al. (2008) set out to confirm via experimental means that NPIs trigger the validation of downward entailing inferences, they found no evidence of a connection between NPI processing and the process of inference validation. This suggests that, while the downward entailingness generalization captures a wide range of facts concerning NPI distribution, it might not be justified after all to assume that this generalization finds a parallel within the actual cognitive mechanisms involved in NPI processing.

#### A Unified Semantic Theory of NPI and NC Constructions

Giannakidou (1999, 2000) provides an alternative semantic account to explain NPI licensing behaviors, and relates them directly to NC. Under her proposal, “NC is nothing more than a subcase of negative polarity” (Giannakidou, 2000, p. 463)^13^. She argues that noun phrases which participate in NC in Greek (a “strict” NC language) are non-negative universal quantifiers that, like NPIs, are sensitive to the veridicality of their surrounding context^14^. Under her theory, these quantifiers must raise to take scope over a sentential negation. The following is an example of NC in Greek, and the corresponding structure (example (23) is her p. 499 ex. (83), and (24) is adapted from p. 500 ex. (90)):


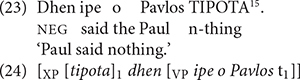
^15^

The structure in (24) shows the phrase *TIPOTA* “n-thing” raising from within the verb phrase to the clause edge, where it marks its scope over the negative marker *dhen*. Crucially, the phrase *TIPOTA* is not itself semantically negative. Since the marker *dhen* contributes the only semantic negation in the structure, the single negation NC reading is derived.

From the perspective of this paper, the importance of Giannakidou’s (1999, 2000) theory is the clear link established between NC and NPI constructions. However, along with theories such as Zeijlstra (2004, et seq.), it is difficult to extend to grammatical systems that generate both NC and DN (e.g., Déprez, 2011; Puskás, 2012; Déprez et al., 2015, among others), including English (e.g., Blanchette and Lukyanenko, 2019). For example, if English negative phrases are NPI-like, then they should not be able to occur in DN constructions. A further prediction is that languages with NC should not have negative phrases appearing with no accompanying clause-bound negative marker, but as Tubau (2016) shows, such sentences coexist in vernacular Englishes alongside NC [see (9) and (10) above], and as we demonstrate below in our experimental results, the same appears to be the case for SE^16^.

#### Strong vs. Weak NPIs

Zwarts (1998) observes within-language diversity in NPI licensing patterns, which serves as the basis for the two syntactic conditions we employ in our experiment. Consider the following examples:

(25) Maria didn’t eat anything for lunch today.
(26) Maria didn’t eat a damn thing for lunch today.
(27) If Maria eats anything for lunch today, she’ll be able to work through the afternoon.
(28) *If Maria eats a damn thing for lunch today, she’ll be able to work through the afternoon.

Sentences (25) through (28) contain the NPIs *anything* and *a damn thing*. While *anything* is acceptable in both the negative context in (25) and the non-negative conditional context in (27), *a damn thing* is only acceptable in the negative context (26), and (28) is unacceptable. Zwarts characterizes this behavior in terms of NPI strength. NPIs such as *a damn thing* are strong, in that they require a strong licensing context such as negation. NPIs such as *anything* are weak, in that, while they are licensed under negation, they may also appear in semantically weaker contexts such as conditionals^17^.

#### A Unified Syntactic Account of NPIs, NC, and DN

Postal (2005) diverges from previous accounts of NPI behavior in proposing that NPIs themselves introduce negation into the structure. Under his theory, there exist two possible underlying structures for NPIs, which Collins and Postal (2014) call “unary NEG” NPIs and “reversals,” and which they propose map onto strong and weak NPIs respectively. The following are Postal’s proposed structures for these two NPI types:


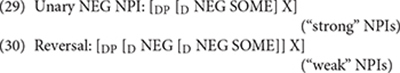


Both structures are noun phrases (DPs) with a negation (NEG) directly modifying an abstract SOME.

Postal (2005) further proposes that NPIs with the forms *anything*, *anybody*, and the like, may have either a unary NEG or a reversal structure. When they occur with the unary NEG structure, the negation that is introduced within the NPI raises to a higher position in the syntax, as follows:


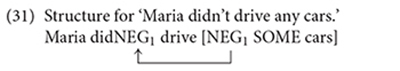


Collins and Postal (2014) propose that the surface form for a structure such as (27) is derived when the lower copy of the negation goes unpronounced and abstract SOME maps to surface form *any*. The structure in (31) thus derives the dependency between the NPI and the higher negation without appeal to semantic licensing.

Note now that the reversal structure in (30) has a second negation. Their proposal is that the outer negation cancels the force of the inner one, yielding a non-negative semantics. This model thus generates the correct truth conditions for sentences such as conditionals, in which NPIs are licensed. For example, in the sentence *If Maria drives any cars, she’ll drive them fast*, the term *any* can be replaced by *some* (or removed entirely) with no change in truth conditions^18^.

Blanchette (2015) uses data from Appalachian vernacular English to show how this system readily extends itself to NC. For an NC sentence like *Maria didn’t drive no cars*, the structure is the same as in (31), except both copies of the negation are spelled out in the phonology, leaving abstract SOME unpronounced. For the DN interpretation (which also exists in Appalachian), the structure simply contains two distinct semantic negations, and there is no NEG raising to a higher position, hence no negative dependency is established:


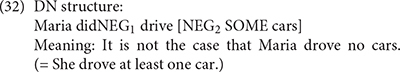


A further benefit of the Postal (2005) and Collins and Postal (2014) system is that it also captures data such as those observed in Tubau (2016), in which negative noun phrases appear variably in concord with a clause mate negative marker, and independently, with no negative clause mate, as in (9) and (10) above. The theory derives these by positing that a unary NEG noun phrase is present in the structure, but the negation remains in its base position and does not undergo raising.

### The Current Study

In light of the English data examined here, a benefit of Postal (2005) and Collins and Postal’s (2014) theory is that it allows for the generation of both NC and DN structures alongside NPI constructions, within the same grammatical system, while previous syntactic and semantic accounts these phenomena do not yet have a clear answer for how all of this might work together. While the current study is not designed to test a particular theory, it does explore the degree to which the same population of speakers treats sentences with overtly negative noun phrases and NPI constructions as parallel, and therefore, the extent to which it is desirable to model them in the same way. We sought to find experimental evidence to support the idea that speakers calculate parallel truth conditions for NC and NPI constructions with negative marker, a “strong” licensing context (and both underlyingly unary NEG structures according to Collins and Postal, 2014 and Blanchette, 2015), and concurrently, whether these same speakers understand that the semantic contributions of the NPI and negative noun phrase yield opposite truth conditions in conditionals, a “weak” and non-negative NPI licensing context (and a context for reversals under Collins and Postal, 2014). As we will show below, the experiment design works because of the nature of the NPI itself. Specifically, when in the scope of a negation, the NPI shares a meaning with the overtly negative noun phrase in NC, but when in the “weak” reversal context of a conditional it takes on the opposite meaning, which is logically non-negative.

## Materials and Methods

### Research Questions

Our experiments were designed to explore similarities and differences between overtly negative noun phrases and NPIs in direct object position under negation, a context for “strong” NPI licensing or unary NEG NPI structures, and under conditionals, a context for “weak” NPI licensing or reversal structures (Zwarts, 1998; Postal, 2005; Collins and Postal, 2014). We asked the following questions:

(i) Do English speakers access parallel meanings for NPI and NC constructions under negation (i.e., contexts for unary NEG structures), despite asymmetries in the acceptability of these constructions?
(ii) Do these same English speakers readily distinguish between the meanings of NPIs and overtly negative noun phrases in “weak” (reversal) licensing contexts, which do not parallel NC?

### Participants

Thirty participants (10 women, 20 men) were recruited through Amazon Mechanical Turk (AMT) for the main experiment, and a further 15 (5 women, 10 men) were recruited for the follow-up. To participate, speakers had to confirm that they were at least 18 and spoke American English natively. Completing the online survey took approximately 30 min, and participants were paid $6 for their time.

All participants had spent most or all of their lives in the US. Their answers to free response questions about cities and regions where they had lived indicated that 17 had spent the majority of their childhoods in the south (including 4 in Florida and 3 in Texas), 9 in the Midwest, 7 in the midatlantic, 6 on the west coast, 1 each in the northeast, great plains and southwest, and that 3 had spent similar amounts of time in two or more regions. Four participants reported familiarity with a language other than English, two heritage language speakers (Chinese, Spanish), and two foreign language learners (Spanish, German).

Participants were between 24 and 72 years old (main study mean = 38.5 years, follow-up study mean = 40.2 years), and the majority had completed either high school (*n* = 9), or a 2-year (*n* = 10) or 4-year college degree (*n* = 16). Of the remaining participants, 5 had completed a graduate degree, 4 had begun a bachelor’s degree, and 1 had begun a graduate degree.

An additional 5 participants, 4 from the main experiment and 1 from the follow-up, completed the task and were paid, but were excluded from the final dataset for failing to achieve 80% accuracy on the catch trials [described below, see (31g)]. These participants gave ratings of 5 or higher (i.e., felicitous) to 4 or more of the 16 fillers that were designed to have infelicitous continuations, or gave ratings of 4 or lower (i.e., unacceptable) to 4 or more of the first clauses of these fillers, despite the fact that these first clauses were unremarkable English sentences. This indicated either that they were not reading carefully, or that they had misunderstood the task.

In a post-survey language questionnaire, participants were asked how likely they and their family and friends were to use NC and NPI constructions to communicate a negative meaning, on a scale from 1 (never) to 7 (always). Ratings were low for use of NC (participants’ median = 1, mean = 1.84; family and friends median = 2, mean = 2.6), and high for use of NPI constructions (participants’ median = 6, mean = 5.9; family and friends median = 6, mean = 6.0). Given the heavy social stigma associated with NC, we interpret these responses with caution, but they suggest that the group of speakers who participated in our experiments can be characterized as primarily non-NC users.

### Materials and Design

We designed two experiments to explore our research questions. The main experiment compared participants’ ratings of three noun phrase types in negative contexts (a context for “strong” NPIs, or unary NEG structures) and in conditionals (a context for “weak” NPIs or reversals). The follow-up experiment further explored the acceptability of negative noun phrases in conditionals, and participants’ interpretation of NPIs in these non-negative contexts. Both experiments included 48 critical sentences and 112 fillers. All sentences contained two clauses, the second of which described a consequence of or context for the first. See Supplementary Appendix A for a full list of items and fillers.

For critical sentences in the main survey, the first clause was either conditional or negative, and the direct object was a DP of one of three types: bare plural (*people, things*), NPI (*anybody, anything*), or negative noun phrase (*nobody, nothing*). Conditional clauses were followed by consequences consistent with a no-negation meaning, and negative clauses were followed by consequences consistent with a single negation meaning. DP type and sentence type were fully crossed within participants such that an individual participant saw 8 items in each of the six conditions, and never saw more than one form of a given item. Half of the items in each condition had animate direct objects (i.e., *people, anybody, nobody*), and half had inanimate objects (i.e., *things, anything, nothing*). Across participants, each item appeared equally in each condition, in a Latin Square design. Example sentences are shown in (33).


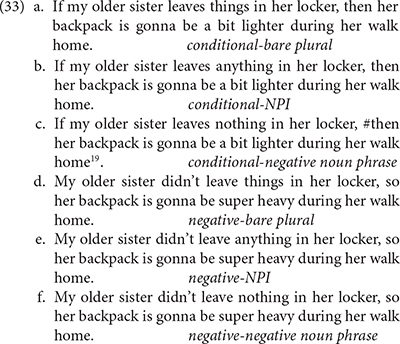
^19^

We were particularly interested in the comparison between NPIs and negative noun phrases in conditional and negative contexts, since this would show us whether speakers calculate parallel truth conditions for NC and NPI constructions in “strict,” unary NEG contexts, and whether these same speakers also calculate opposite truth conditions when these noun phrase types appear the “weak” reversal context of conditionals. Constructions with a bare plural, which have the same truth conditional meaning as the NPIs in these sentences but no linguistic dependency, were employed as a control.

Critical sentences in the follow-up survey were derived from those in the main survey, by pairing the conditional first clauses with the single-negation continuations, as shown in (34). This was intended to render the negative noun phrases fully felicitous in the conditional sentences, and the NPIs and bare plurals infelicitous. Because there were only three conditions, participants saw twice as many sentences per condition as in the main study, and only half as many participants were needed to obtain the same number of observations per condition.


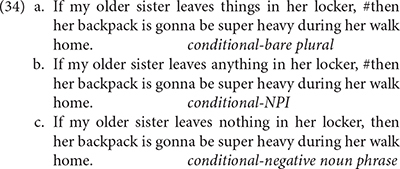


Fillers were identical for the two surveys and were designed with the same two-clause structure as critical sentences. They included a variety of features intended to blend with the critical items, including several different subordinating conjunctions, universally quantified direct objects, and a single negated auxiliary without quantified or bare plural direct objects, as shown in (35). Of the 112 fillers, 96 were designed to have felicitous continuations (35a-f), and the remaining 16 were designed to be fully acceptable but have infelicitous continuations (35g). This created a similar proportion of infelicitous continuations in the filler items as we predicted there would be in the critical items (1/7 and 1/6 respectively). The 16 “mismatch” fillers served as catch trials and allowed us to exclude participants who had misunderstood the task or were not attending it carefully (*n* = 5, see section Participants).


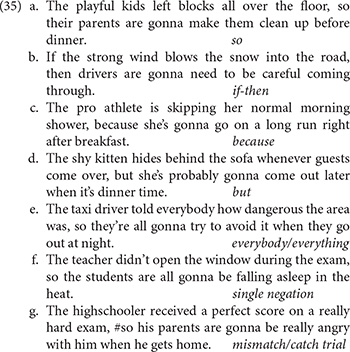


### Procedure

Upon selecting the survey, AMT workers were directed to a Qualtrics survey link. They first read and acknowledged an informed consent statement, then proceeded to the survey^20^. For each item in the survey, participants were asked to first judge the naturalness (acceptability) of the first clause, and then judge the plausibility (felicity) of the second clause^21^. The targeted clause was bolded during the relevant judgment, but the entire sentence was visible throughout the trial. Both judgments were on a 7-point Likert scale, with endpoints labeled “completely natural” (7) and “completely unnatural” (1) for the acceptability rating, and “consequence makes total sense” (7) and “consequence makes zero sense” (1) for the felicity rating.

The survey was preceded by four practice trials with feedback, to familiarize participants with the task. Of the 4 practice trials, two had low acceptability first clauses (glaring word order errors), and two had high acceptability first clauses. This was crossed with plausibility of the consequence, to demonstrate the independence of the two judgments.

The body of the survey included the 112 fillers and 48 critical items presented in a fully random order and was followed by a short debriefing and language history questionnaire. Upon completion of the survey, participants were given a code to enter into the AMT interface in order to get their payment.

### Analyses

Both acceptability and felicity ratings were on a 7-point Likert scale, and were therefore analyzed using ordinal rather than linear regression techniques (Liddell and Kruschke, 2018). All models were cumulative link mixed models, fit using the *clmm()* function of the *ordinal* package (version 2019.4-25; Christensen, 2019) in R (version 3.6.0; R Core Team,, 2019) and a probit link function.

This analysis technique differs in several ways from other common approaches to analyzing Likert data. Most importantly, in contrast to linear modeling techniques, ordinal modeling does not make the assumption that participants treat the ratings as equally spaced. That is, ordinal modeling allows for the possibility that participants will, for instance, be particularly hesitant to give the minimum rating, effectively making the distance between 1 and 2 larger than the distance between 2 and 3. Second, raw ratings are entered into the model, rather than z-scored ratings. Z-scoring serves two purposes when analyzing ratings using linear models: to make the measure more continuous and therefore more appropriate for a non-ordinal analysis, and to factor out between-participant variation. The use of ordinal analysis obviates the need for continuity, and mixed model approaches, whether linear or ordinal, take between-participant variation into account using random effects.

When interpreting model output, note that estimates are threshold changes in terms of shared standard deviation. Thus, while they are not readily interpretable as predicted change in score or probability (as might be the case in a well-coded linear model), they are readily comparable to each other within a model: an estimate of 3 indicates that a factor has twice as large an influence on the thresholds as a factor with an estimate of 1.5.

For other linguistic studies applying cumulative link mixed models to Likert scale judgment data, see Clifton et al. (2019), Fekete et al. (2018), and Scontras et al. (2017).

## Results

To explore the relationship between participants’ acceptance of English NC and their ability to interpret it as truth conditionally equivalent to negative NPI constructions, we compared participants’ acceptability ratings of three types of direct object (overtly negative noun phrases, NPIs, bare plurals) in negative and conditional sentences. Each initial clause was followed by a second clause that, for the negative sentences, was compatible with a single negation reading, and for the conditional contexts was compatible with a no-negation reading. We predicted that participants would rate all first clauses as relatively acceptable except for the negative noun phrase in a *negative* sentence, i.e., the stigmatized NC construction. We furthermore predicted that they would rate the consequence as highly felicitous for all second clauses except the negative noun phrase in a *conditional* sentence, which is incompatible with the meaning expressed in the consequence.

In the follow-up survey, we paired the single-negation compatible consequences with the conditional first clauses [see (30)] in order to confirm that negation is not uniformly less acceptable in conditionals, and that participants treat NPIs and negative noun phrases as opposites in non-negative conditional statements, in contrast to the negative contexts in the main study where we predicted they would be treated as syntactic variants.

Crucially, we predicted a disconnect between participants’ acceptability ratings for NC sentences, and their felicity ratings for the single negation continuations in the main study, which would be instantiated as low acceptability but high felicity ratings for negative sentences with negative noun phrases. High felicity ratings would indicate that participants readily achieved the intended reading of the NC construction, and would suggest that low acceptability ratings are likely more the result of social pressure than the speaker’s inability to generate the structure.

### The Main Experiment

**FIGURE 1:**
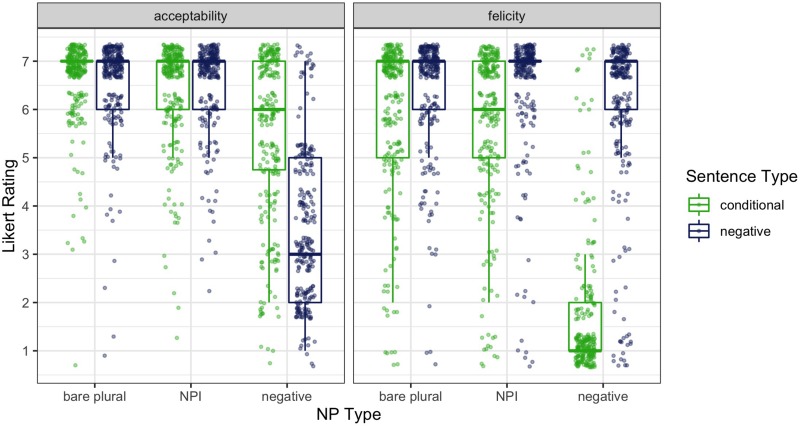
Raw acceptability and felicity judgments for the main survey on a 7-point Likert scale, with box plots showing overall quartiles and median. Values for conditional sentences are shown in light green, and for negative sentences in dark blue.

Figure 1 shows jittered raw ratings of sentence acceptability (left panel) and consequence felicity (right panel), along with boxplots to help summarize the distribution. The most striking pattern is the predicted reversal of the sentence type effect on negative noun phrases across the two panels. Negative noun phrases were rated as relatively unacceptable (median = 3) in negative contexts (the stigmatized NC construction), but their continuations, consistent with the single negation NC reading, were rated as highly felicitous (median = 7). In contrast, negative noun phrases in conditional sentences were rated as acceptable (median = 6), but their no-negation continuations were rated as infelicitous (median = 1). That is, participants appear to have rated stigmatized NC constructions as unacceptable, but readily generated the single negation interpretation necessary to make the consequence felicitous^22^. This supports the hypothesis that these constructions are part of the participants’ grammars, but that their acceptability rating is heavily influenced by social pressure, and therefore serves as a poor diagnostic for grammaticality.

Other patterns visible in the graph include very high acceptability ratings for both bare plurals and NPIs in both conditional and negative sentences (all medians = 7), with the most consistently high acceptability ratings for bare plurals in conditional sentences, and generally high felicity ratings for consequences following bare plurals and NPIs (median = 6 for conditional-NPI, 7 elsewhere). Also note that there is more spread in the generally low ratings for negative NPs in the negative sentences (median = 3) than one might expect for something truly unacceptable. Compare, for instance, the consistent, very low felicity ratings (median = 1) for the truly infelicitous continuations, following conditional sentences with negative noun phrases. We return briefly to this variability in the discussion.

To explore these patterns statistically, we fit separate cumulative link mixed models for acceptability ratings and felicity ratings (see section Analyses). For both models, predictor variables were the two-level factor sentence type (conditional, contrast code -0.5 vs. negative, contrast code 0.5), and the three-level factor NP type, coded as two Helmert contrasts, the first comparing negative noun phrases to NPIs and bare plurals together (“negative-other,” negative noun phrases, 0.67 vs. NPIs and bare plurals, both -0.33), and the second comparing NPIs to bare plurals (“NPI-bare,” NPIs, -0.5 vs. bare plurals, 0.5, negative NPs, 0), as well as the interactions of sentence type and the NP type contrasts. The model included random intercepts for item and participant and the random slopes of sentence type by item and of NP type, sentence type and their interaction by participant.

#### Acceptability

Model results are shown in Table 1. All main effects were reliable, as was the key interaction of sentence type and the negative-other NP type contrast [all LR(1) > 4, all *p* < 0.05]. This reliable interaction is consistent with our expectation that negative noun phrases in negative contexts would be treated as particularly unacceptable.

**TABLE 1:** Model results for acceptability ratings in the main survey.

**Effect**	**Estimate**	**se**	***z***	**LR (1)**	***p***
**NP type**					
negative-other	–2.40	0.20	–11.78	57.85	< 0.00001
NPI-bare	0.33	0.16	2.03	4.05	0.044
**Sentence type**	–0.57	0.13	–4.21	14.27	0.0002
**Sentence type × NP type**					
negative-other	–1.75	0.25	–7.14	33.26	< 0.00001
NPI-bare	–0.52	0.31	–1.68	2.65	0.10

**All p-values were obtained using likelihood ratio tests.**

Planned comparisons further explored the key interaction and supported this conclusion. Three models, identical to the main model except for their contrast codes, were conducted to examine the simple main effects of both NP type contrasts in conditional and in negative sentences, and to examine the simple main effect of sentence type on the acceptability of negative noun phrases. These models revealed that negative noun phrases were less acceptable than NPIs and bare plurals in negative sentences [*b* = −3.27, se = 0.28, LR(1) = 57.65, *p* < 0.00001] and somewhat less acceptable (note the much smaller estimate) in conditional sentences [*b* = −1.52, se = 0.19, LR(1) = 38.5, *p* < 0.00001]. The interaction in the main model indicates that this difference was reliably larger for negative sentences than conditionals, and a follow-up comparison confirms that negative noun phrases in negative sentences (i.e., NC constructions) were reliably less acceptable than in conditional sentences [*b* = −1.73, se = 0.21, LR(1) = 38.82, *p* < 0.00001]. Intriguingly, NPIs were also very slightly but reliably less acceptable than bare plurals in conditional sentences [*b* = 0.59, se = 0.21, LR(1) = 8.36, *p* = 0.004], but not in negative sentences [*b* = 0.06, se = 0.24, LR(1) = 0.07, *p* = 0.79], perhaps reflecting the additional processing load incurred by the interaction between the NPI and the conditional. We discuss this further below.

#### Felicity

For felicity ratings, the continuations of the conditional sentences with negative noun phrases were predicted to be infelicitous, which should result in a reliable interaction between sentence type and the negative-other NP type contrast. This prediction was supported by the model results, shown in Table 2. All main effects and the interaction of sentence type with the negative-other NP type contrast were statistically reliable [all LR(1) > 7, all *p* < 0.01]. The interaction of the negative-other contrast and sentence type supports our prediction that negative noun phrases in conditional contexts would be treated as particularly infelicitous.

**TABLE 2:** Model results for felicity ratings in the main survey.

**Effect**	**Estimate**	**se**	***z***	**LR (1)**	***p***
**NP type**					
negative-other	–1.64	0.13	–12.69	57.46	< 0.00001
NPI-bare	–0.05	0.10	–0.49	0.25	0.62
**Sentence type**	1.51	0.15	9.91	51.02	< 0.00001
**Sentence type × NP type**					
negative-other	2.26	0.29	7.85	43.09	< 0.00001
NPI-bare	–0.51	0.19	–2.65	7.37	0.007

**All p-values were obtained using likelihood ratio tests.**

This primary model was again followed by further analyses to explore the interactions in the data. These revealed a reliable effect of the negative-other NP type contrast in both conditional [*b* = -2.77, se = 0.20, LR(1) = 64.57, *p* < 0.00001] and negative sentences [*b* = -0.51, se = 0.19, LR(1) = 5.84, *p* = 0.02]. The former supports the predicted interaction, and the latter indicates that while negative noun phrases were felicitous under negation (with median acceptability of 6), they were reliably less felicitous than NPIs and bare plurals. Further supporting the predicted interaction, we found that continuations of conditional sentences with negative noun phrases were reliably less felicitous than continuations of negative sentences with negative noun phrases [*b* = 3.01, se = 0.30, LR(1) = 46.58, *p* < 0.00001]. This indicates that participants reliably distinguished between NC sentences which made the continuation felicitous, and conditional *if*-clauses which did not.

Again, intriguingly and consistent with the overall interaction between sentence type and the NPI-bare contrast, there were differences between NPIs and bare plurals. There was a reliable effect of the NPI-bare contrast in negative sentences [*b* = -0.30, se = 0.16, LR(1) = 4.13, *p* = 0.04] and a marginal one in the opposite direction in conditional sentences [*b* = 0.20, se = 0.11, LR(1) = 2.90, *p* = 0.09]. That is, continuations were reliably rated as more felicitous for bare plurals in conditionals and for NPIs in negative sentences, perhaps reflecting a greater ease of processing NPIs in negative (“strict,” or unary NEG) contexts than in non-negative conditional (“non-strict,” or reversal) contexts.

### The Follow-Up Experiment

**FIGURE 2:**
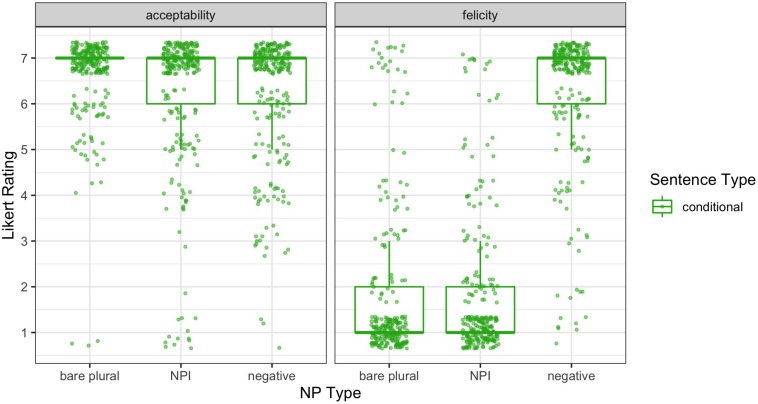
Raw acceptability **(left panel)** and felicity ratings **(right panel)** for the follow-up survey on a 7-point Likert scale, with box plots showing overall quartiles and median.

One possible explanation for the pattern of felicity ratings in the main study is that the consequences of conditional sentences with negative direct objects were rated as infelicitous at least partly because negation is difficult to process, and this was exacerbated by the presence of the conditional. The follow-up survey was designed to confirm first that sentences with negative noun phrases were not inherently less felicitous under conditionals, and further, to confirm that speakers understand when negative noun phrases are equivalent in meaning to NPIs and when they are not. Figure 2 shows participants’ raw acceptability and felicity ratings for the sentences in the follow-up survey. Acceptability was very high across all NP types (all medians = 7), and felicity of the single negation consequence was rated as very low for the bare plural and NPI sentences (medians = 1), and very high for the negative NP sentences (median = 7).

We again fit cumulative link mixed models of acceptability ratings and of felicity ratings, this time with a single fixed effect predictor, the three-level Helmert-coded factor NP type. The models had random intercepts for participants and items, and random slopes for NP type by participant. For acceptability ratings, the model revealed only a marginal main effect of the NPI-bare NP type contrast (model results are shown in Table 3), reflecting the slightly higher ratings for bare plurals relative to NPIs and replicating the pattern found in follow-up analyses in the main study. There was no reliable decrease in acceptability for negative noun phrases as compared to the other NP types.

**TABLE 3:** Model results for acceptability ratings in the follow-up survey.

**Effect**	**Estimate**	**se**	***z***	**LR (1)**	***p***
**NP type**					
negative-other	–0.31	0.27	2.08	1.21	0.27
NPI-bare	0.51	0.25	–1.15	3.53	0.06

**All p-values were obtained using likelihood ratio tests.**

For felicity ratings, there was no reliable difference between bare plurals and NPIs, but there was a reliable difference between negative NPs and the other NP types (model results are shown in Table 4). This again confirms that in conditional sentences, negative (or unary NEG) noun phrases contribute a negative meaning, rendering the single-negation compatible continuation felicitous, while NPIs (here, reversals) contribute a meaning truth conditionally equivalent to non-negative bare plurals.

**TABLE 4:** Model results for felicity ratings in the follow-up survey.

**Effect**	**Estimate**	**se**	***z***	**LR (1)**	***P***
**NP type**					
negative-other	2.67	0.26	10.43	33.07	< 0.00001
NPI-bare	–0.01	0.13	–0.09	0.01	0.93

**All p-values were obtained using likelihood ratio tests.**

## Discussion

Our main experiment involved two comparisons, one which compared negative noun phrases, NPIs, and bare plural controls under negation, and another which compared these same elements under non-negative conditionals. We asked whether speakers would calculate parallel truth conditions for NPIs and overtly negative noun phrases under negation (a context for unary NEG structures), and whether these same speakers would calculate opposite truth conditions for these words in non-negative conditionals (a context for reversal structures). We first discuss the comparison which involved negative dependencies.

### Negative Contexts

Comparison of the three argument types in syntactically negative contexts revealed an asymmetry which can inform our understanding of the relationship between NC and NPI constructions: Participants’ acceptability ratings of socially stigmatized NC constructions were low, and their felicity ratings of consequences which correspond to the NC interpretation for these same constructions were high. Furthermore, while NC and NPI constructions were rated on opposite sides of the scale in acceptability, with NC on the low side and NPIs on the high side (and similar to bare plural controls), the consequences of all construction types were given relatively high felicity ratings.

Regarding the asymmetry between NC acceptability and felicity, we note that this finding both supports and complements previous work which compared NC with DN, its truth conditional opposite (Blanchette, 2017; Blanchette et al., 2018; Blanchette and Lukyanenko, 2019). In these studies, preceding context was employed to elicit an NC or a DN reading for sentences a subset of which were parallel to the critical sentences presented here. Speakers were shown, through a variety of measures including naturalness ratings, forced choice judgments of meaning, acoustic production and perception, and online reading times, to reliably prefer the NC over the DN interpretation for these items.

In the current study, there were no DN interpretations elicited from speakers during the course of the experiment, and given that participants reliably judged the single negation consequence of NC constructions as felicitous (which would have been infelicitous on a DN reading), we can assume that, at least for the most part, participants did not generate DN meanings for the items with two syntactic negations. Other differences between this study and those previous studies include the fact that participants judged NPI and bare plural sentences as well as NC, and that their judgments were made on the basis of the NC interpretation’s felicitousness as determined by a following consequence, as opposed to a preceding context. In the context of previous studies in which speakers reliably prefer NC over DN interpretations in a range of measures, the fact that a distinct design led to similar results thus further confirms the robustness of speakers’ readiness to interpret NC constructions as singly negative, and provides complementary support for the hypothesis that speakers who do not accept NC nevertheless have grammatical knowledge of it.

Regarding the interactional aspect of the asymmetry in negative contexts, in which NC acceptability and felicity were at opposite sides of the scale, while NPI (and bare plural) acceptability and felicity were on the same side, this shows that participants readily accessed the same truth-conditional meaning for all three NP types under negation, despite reliable differences in their acceptability. It should be noted that there was in fact a small but statistically reliable difference between NPI and bare plural felicity in negative contexts on the one hand, and NC felicity on the other. We believe this difference is best explained as a carryover effect of the strong unacceptability of NC. This is particularly likely since, as explained in the methods section, participants still had the critical sentence in view when judging the consequence.

The interaction between NC and NPI constructions in negative contexts also illustrates a more general methodological point, namely, that examining acceptability in isolation from meaning can obscure speaker knowledge of a construction type (especially where that construction type is socially stigmatized). In this case, the social stigma associated with English NC appears to be a primary force shaping speakers’ acceptability ratings. Yet despite the strength of this social stigma, participants drew a clear distinction between the acceptability of NC and its meaning in context. NC thus provides an example of a construction type for which binary or overall acceptability and interpretation are unrelated. We extend this to suggest that NC also provides an example of a construction type for which overall acceptability and *grammaticality* are unrelated, and participants are able to interpret NC structures because their grammars generate them. This means that, in the case of NC, the traditional direct link between acceptability and grammaticality fails. Below we discuss some theoretical implications of participants’ readiness to assign the same meaning to NC and NPI constructions, despite their distinct acceptability status.

To conclude this subsection, we note that there was substantially more spread in the negative sentence-negative noun phrase (i.e., NC) acceptability ratings than what might be expected for something that is outright ungrammatical (e.g., sentences with glaring word order violations such as *Up the bike the woman the hill rode*). The median response for NC sentences is 3, and observing the individual data points in Figure 1, we see that there are also many 4s and 5s. Thus, while overall acceptability is significantly lower for these NC constructions than for their prescriptively correct variants, these middling acceptability ratings may hint at their hypothesized grammaticality. Another possibility is that, because a large proportion of the sentences within the experiment were acceptable, participants were more inclined to provide slightly higher ratings even for the least acceptable sentences. The latter interpretation maintains the conclusion that there is no relation between English NC acceptability and grammaticality, while the former suggests some potential overlap.

### Conditional Contexts

Items where the NPIs, overtly negative noun phrases, and bare plurals appeared under conditional contexts displayed two clear additional asymmetries beyond the ones found in negative contexts. In the main experiment, the clearest asymmetry was again interactional in nature, between the NPIs and bare plurals on the one hand, and the overtly negative noun phrases on the other. These were all relatively acceptable, with mean scores well above the middle of the scale, but in the main study, the contexts were designed to make the NPIs and bare plurals felicitous and the overtly negative noun phrases infelicitous. Unsurprisingly, participants responded in reliable fashion to this experimental manipulation, rating consequences following *if* clauses with overtly negative noun phrases as extremely low, despite the relative acceptability of the *if* clause itself. Viewed alongside the behavior of NC and NPI constructions in negative contexts, what this asymmetry shows is that the same participants who understood that negative noun phrases and NPIs are truth conditionally equivalent in negative contexts (i.e., contexts for unary NEG structures) readily reversed the truth condition for NPIs in non-negative (i.e., reversal) contexts.

The follow-up experiment was designed to inform the results of the main experiment, and to provide a more complete picture of speakers’ understanding of where contexts for NPIs and overtly negative noun phrases do and do not overlap. Reversing the truth conditions for the consequence from the main experiment, we expected that the non-negative NPI (a reversal structure), and not the (unary NEG) negative noun phrase, would be infelicitous. Participants again behaved as predicted, rating consequences of NPI and bare plural *if* clauses at the very low end of the scale, and consequences of overtly negative noun phrases at the high end. This allows us to point to the consequence as the source of infelicity for the negative noun phrase in conditionals in the main experiment. Additionally, it confirms that speakers understand when the meaning of an NPI is equivalent to an overtly negative noun phrase which participates in concord, and when it is not.

Before turning to theoretical implications, we note an additional asymmetry that our experiment was not explicitly designed to reveal: Though acceptable overall, overtly negative noun phrases were slightly less acceptable than NPIs and bare plurals in the main experiment conditional contexts. One potential explanation for this is that negation makes things more difficult to process (e.g., Ferguson et al., 2008), thus degrading acceptability, and further, that participants prefer a more focalized information status for negative objects with no preceding negative marker (e.g., Childs, 2017; Palacios Martínez, 2017). Note, however, that when the consequence for *if* clauses with an overtly negative noun phrase object was made felicitous, as in the follow-up experiment, the median acceptability of *if* clauses with NPIs and those with overtly negative noun phrases was nearly identical. It is therefore more likely that the infelicity of the consequence carried over here in the reverse direction, degrading the acceptability of the *if* clause where the object was overtly negative. This conclusion is supported by the fact that NPI acceptability in *if* clauses was on par with negative noun phrase acceptability in the follow-up experiment. Interestingly, this degradation effect did not apply to the bare plurals in the follow-up experiment. This suggests a potentially interesting conclusion that the source of this degradation is the negative dependency itself, suggesting that the cost of processing this dependency impacts acceptability ratings. Alternatively, it might be the case that the presence of heavily stigmatized NC in the main experiment served to degrade participants’ acceptability judgments of all sentences with negative noun phrases. We leave this matter for future research.

### Theoretical Implications

One explanation for the fact that participants gave similar felicity judgments for the NC and NPI constructions in negative or “strict” contexts is that their grammars represent NC and NPI constructions as syntactic variants with the same underlying form. This explanation finds its theoretical basis in Postal (2005) and Collins and Postal’s (2014) analysis of NPI constructions, and Blanchette’s (2015) extension of this proposal to English NC, described above. Under this theory, the grammar of the negative NPI and the NC constructions in this experiment involve the raising of a negation from the object noun phrase to a higher clausal position, generating a syntactic dependency between the negative marker and the object. The only difference between the two constructions is at the level of phonological spell out: In the one that surfaces as an NPI construction, the negation is unpronounced (and an abstract SOME spells out as *any*), whereas the NC construction involves spell out of both negations (and a silent abstract SOME).

The process governing the spell out of the lower negation in unary NEG structures may be grammatical in nature, where SE grammars have a constraint that prohibits them from pronouncing the lower negation which is absent from vernacular varieties, or it may be a purely socially governed phenomenon which over time has been conventionalized in SE, with the effect of masking a direct underlying grammatical connection between these two construction types. Whether the differences between these two surface forms are derived by grammatical or social pressures, a plausible explanation for our results is that speakers generated the same negative dependency in both the NC and the NPI constructions in negative contexts, and this was reflected in their felicity judgments. Concurrently, their clear intuitions about the opposite meanings of negative noun phrases and NPIs in conditionals, a “weak” licensing context, supports the hypothesis that they also have two distinct underlying representations for NPIs, a unary NEG structure and a semantically non-negative reversal, and they select the item analogous to the reversal structure for these conditional contexts.

We can also view our results in light of Tubau’s (2016) theory of English NC. The extension would be similar to that of Collins and Postal (2014) in the sense that it would also assume speakers have two lexical entries for the same word, except that, instead of having two entries for *any*-NPIs, there would be two distinct entries for overtly negative noun phrases, one of which appears in NC constructions, and one of which appears in conditionals. We would then need to extend the theory further to account for the behavior of NPIs, and specifically, to explain not just the dependencies involved in these, but also, why they overlap in meaning with NC constructions in negative contexts, but contribute a meaning that reverses the truth conditions for the negative noun phrase in conditionals.

With regard to purely semantic theories of NPI licensing, in addition to finding experimental evidence for a parallel to the calculation of downward entailing inferences (Ladusaw, 1979), or (non-)veridicality (Giannakidou, 1998, 1999) in processing, we would now also need to explore whether the dependency established in NC, coupled with the now well-established observation that NC and DN may coexist in a single system, can also be explained by these theories. We set these questions, and the design of more targeted experiments which can tease apart these theories of grammar, aside for future work.

## Conclusion

The experiments we reported here revealed asymmetries in the acceptability and felicity of NC and NPI constructions. We have provided evidence that speakers understand when the truth conditions for NC and NPI constructions overlap, and when they do not. The results have both methodological and theoretical implications. On the methodological side, they demonstrate a clear case where there is no straightforward causal link between acceptability and grammaticality, and concurrently how judgments of meaning can inform theories of grammar in cases where acceptability judgments fail. On the theoretical side, they show how the set of facts that grammatical theories should be capable of modeling within a single system includes NC and NPI constructions, and in the context of previous studies, also DN. We further discussed how the system in Postal (2005) and Collins and Postal (2014), and its extension in Blanchette (2015), provides one such theory, while other existing theories do not yet explicitly capture the full range of facts.

## Data Availability Statement

The datasets generated for this study are available on request to the corresponding author.

## Ethics Statement

Ethical review and approval were obtained for the study on human participants in accordance with the local legislation and institutional requirements, and the study was determined to be exempt from continuing review. Written informed consent to participate in this study was not required in accordance with the national legislation and institutional requirements.

## Author Contributions

Both authors made substantial contributions to the development of this work, including experimental design, data collection and analysis, drafting and revising the manuscript, and agreed to be accountable for all aspects of the work.

## Conflict of Interest

The authors declare that the research was conducted in the absence of any commercial or financial relationships that could be construed as a potential conflict of interest.

## Footnotes

1 Typical of Romance languages like Catalan, French, Italian, Spanish, and Brazilian Portuguese (e.g., Zanuttini, 1997; Déprez, 2000; Herburger, 2001; De Swart and Sag, 2002; Prieto et al., 2013; Agostini and Schwenter, 2018), NC can also be found many other languages including Afrikaans (Biberauer and Zeijlstra, 2012), Bavarian (Bayer, 1990), Hungarian (Puskás, 2012), West Flemish (Haegeman and Zanuttini, 1996), Serbian/Croation (Progovac, 1994), and others.

2 See Auwera and Alsenoy (2016, p. 483) on the frequency of negative structure types across languages.

3 See Giannakidou (2000) for a semantic account, discussed further below.

4 Following AAPCAppE citation conventions, tokens are followed by the corpus, subcollection, and speaker initials, along with a numerical token identifier.

5 We follow Hudley and Mallinson (2010) in employing the term standardized English, as opposed to the more common “Standard” English, to acknowledge the agency of prescriptive forces in the standardization process, which excludes variants not because of their lack of systematicity, but rather, because of the identities of the speaker groups who do and do not use them.

6 In a study that compares children and adults, Thornton et al. (2016) find that Australian English-speaking adults reliably prefer DN readings over NC readings, in contrast with children, who reliably prefer NC. Children’s judgments were elicited in spoken conversation with a puppet, while adults judgments were collected in written form, which suggests that the comparison is not entirely valid. See Blanchette and Lukyanenko (2019) for further discussion of this.

7 Blanchette (2017) also examines Negative Auxiliary Inversion (NAI) constructions such as the following, in which the negative noun phrase is also in a subject position (for more on NAI see, e.g., Weldon, 1994; Labov, 1972; Green, 2014):

(i) Didn’t nobody see me.
‘Nobody saw me.’

The specifics are beyond the scope of this paper to discuss, but the general conclusion is that SE speakers prefer NC interpretations for sentences in which the negated auxiliary precedes (and c-commands) the negative noun phrase.

8 Vernacular Englishes also optionally instantiate the “strict” NC pattern (Labov, 1972; Wolfram and Christian, 1976; Tubau, 2016; among others).

9 Henry (1995) notes that constructions such as this are possible in Belfast English.

10 Sentence (14) is argued to be unacceptable because it does not meet the c-command requirement for NPIs and their licensors. We discuss this further below.

11 The term “downward entailing” is used synonymously with “monotone decreasing” (Barwise and Cooper, 1981).

12 For reasons of space and lack of immediate relevance we set aside here and throughout instances of “free choice *any*,” as in the following example:

(i) Maria drove any car she wanted.

For a semantic account of free choice *any* (see e.g., Dayal, 1995). For a syntactic account (see Collins and Postal, 2014, p. 43).

13 See Herburger (2001, p. 295) for a similar conclusion.

14 A detailed summary of Giannakidou’s (1999) theory of NPI licensing as veridicality sensitivity is beyond the scope of this paper, but see Giannakidou (2000) and Liu (2019) for this, and see Liu (2019) for an experimental investigation of NPI licensing in conditionals which can be taken to support this theory.

15 Capital letters denote emphasis, which according to Giannakidou (2000) is the property which distinguishes negative universal quantifiers from existentials.

16 See also Agostini and Schwenter (2018) for corpus and experimental evidence of this phenomenon in Brazilian Portuguese.

17 See Gajewski (2011) for a proposal in which NPI strength is explained by appeal to (non-)sensitivity to non-truth conditional aspects of meaning.

18 Collins and Postal (2014, Chapter 8) derive the surface forms of reversal NEG structures by proposing a system of “NEG deletion” which involves a relationship between the inner and the outer NEG, and the outer NEG and a “NEG deleter” that structurally precedes it. The process of NEG deletion removes both negations from the phonological output.

19 The # symbol marks infelicity. We include it here for expository purposes. It was not included in the actual experiment.

20 This survey was conducted under the supervision of the Penn State IRB, which deemed it to be minimal risk and therefore exempt from requirements for written documentation of informed consent. Participants indicated their understanding of the consent document and willingness to participate by simply continuing with the survey.

21 Previous studies have similarly elicited two separate responses of a different nature for a single item. See for example, Blanchette et al. (2018) and Li et al. (2019).

22 We are currently collecting data in a parallel eye-tracking study. Tracking participants’ eye-movements as they read these same stimuli will allow us to more directly investigate just how readily this interpretation is generated.
