# Supplementary material for: Asymmetries in the Acceptability and Felicity of English Negative Dependencies: Where Negative Concord and Negative Polarity (Do Not) Overlap
Source: Front Psychol. 2019 Nov 12;10:2486. doi: 10.3389/fpsyg.2019.02486 (PMC6861449; doi:10.3389/fpsyg.2019.02486)
Supplement: Supplementary file 1 [file Presentation_1.pdf]

Appendix A. Stimulus Items.

The critical sentences for the follow-up survey are derived, with a few minor changes, from the conditional sentences in the main survey: For the follow-up survey the conditional first clauses were simply paired with the continuation from the negative first clauses. Both surveys shared the same set of 112 filler sentences, 16 of which had infelicitous continuations and were used as catch trials. The sentences used in the study are all presented below.

Critical sentences

Table A.1 Critical sentences from the main survey. Each participant saw only one version of each item, in a Latin Square design. This resulted in 6 lists.

Item	First Clause	Continuation	Trial Type
1	If the loud music bothers anybody during dinner,	then the friends are gonna leave the bar after they've eaten.	*conditional-NPI*
	If the loud music bothers people during dinner,	then the friends are gonna leave the bar after they've eaten.	*conditional-bare plural*
	If the loud music bothers nobody during dinner,	then the friends are gonna leave the bar after they've eaten.	*conditional-negative NP*
	The loud music didn't bother anybody during dinner,	so the friends are gonna stay for a drink after they've eaten.	*negative-NPI*
	The loud music didn't bother people during dinner,	so the friends are gonna stay for a drink after they've eaten.	*negative-bare plural*
	The loud music didn't bother nobody during dinner,	so the friends are gonna stay for a drink after they've eaten.	*negative-negative NP*
2	If my favorite aunt brings anybody to the cookout,	then I bet she is gonna be entertained the entire evening.	*conditional-NPI*
	If my favorite aunt brings people to the cookout,	then I bet she is gonna be entertained the entire evening.	*conditional-bare plural*
	If my favorite aunt brings nobody to the cookout,	then I bet she is gonna be entertained the entire evening.	*conditional-negative NP*
	My favorite aunt didn't bring anybody to the cookout,	so I bet she is gonna end up bored the entire evening.	*negative-NPI*
	My favorite aunt didn't bring people to the cookout,	so I bet she is gonna end up bored the entire evening.	*negative-bare plural*
	My favorite aunt didn't bring nobody to the cookout,	so I bet she is gonna end up bored the entire evening.	*negative-negative NP*
3	If the store manager fires anybody for late arrival,	then the workers are gonna be real careful about being prompt.	*conditional-NPI*
	If the store manager fires people for late arrival,	then the workers are gonna be real careful about being prompt.	*conditional-bare plural*
	If the store manager fires nobody for late arrival,	then the workers are gonna be real careful about being prompt.	*conditional-negative NP*
	The store manager didn't fire anybody for late arrival,	so the workers are gonna get careless about being prompt.	*negative-NPI*

	The store manager didn't fire people for late arrival,	so the workers are gonna get careless about being prompt.	*negative-bare plural*
	The store manager didn't fire nobody for late arrival,	so the workers are gonna get careless about being prompt.	*negative-negative NP*
4	If the excited kid follows anybody from her group,	then she is probably gonna find her way in the science museum.	*conditional-NPI*
	If the excited kid follows people from her group,	then she is probably gonna find her way in the science museum.	*conditional-bare plural*
	If the excited kid follows nobody from her group,	then she is probably gonna find her way in the science museum.	*conditional-negative NP*
	The excited kid didn't follow anybody from her group,	so she is probably gonna lose the others in the science museum.	*negative-NPI*
	The excited kid didn't follow people from her group,	so she is probably gonna lose the others in the science museum.	*negative-bare plural*
	The excited kid didn't follow nobody from her group,	so she is probably gonna lose the others in the science museum.	*negative-negative NP*
5	If the piano student follows anybody at the rehearsal,	then she is probably gonna be just fine during the performance.	*conditional-NPI*
	If the piano student follows people at the rehearsal,	then she is probably gonna be just fine during the performance.	*conditional-bare plural*
	If the piano student follows nobody at the rehearsal,	then she is probably gonna be just fine during the performance.	*conditional-negative NP*
	The piano student didn't follow anybody at the rehearsal,	so she is probably gonna be really lost during the performance.	*negative-NPI*
	The piano student didn't follow people at the rehearsal,	so she is probably gonna be really lost during the performance.	*negative-bare plural*
	The piano student didn't follow nobody at the rehearsal,	so she is probably gonna be really lost during the performance.	*negative-negative NP*
6	If the six-year-old fools anybody with her story,	then her family is gonna doubt that she broke the dinner plate.	*conditional-NPI*
	If the six-year-old fools people with her story,	then her family is gonna doubt that she broke the dinner plate.	*conditional-bare plural*
	If the six-year-old fools nobody with her story,	then her family is gonna doubt that she broke the dinner plate.	*conditional-negative NP*
	The six-year-old didn't fool anybody with her story,	so her family is gonna know that she broke the dinner plate.	*negative-NPI*
	The six-year-old didn't fool people with her story,	so her family is gonna know that she broke the dinner plate.	*negative-bare plural*
	The six-year-old didn't fool nobody with her story,	so her family is gonna know that she broke the dinner plate.	*negative-negative NP*
7	If the busy hosts forget anybody before the party,	then they're probably gonna run out of food to feed the guests.	*conditional-NPI*
	If the busy hosts forget people before the party,	then they're probably gonna run out of food to feed the guests.	*conditional-bare plural*
	If the busy hosts forget nobody before the party,	then they're probably gonna run out of food to feed the guests.	*conditional-negative NP*

	The busy hosts didn't forget anybody before the party,	so they're probably gonna have enough food to feed the guests.	*negative-NPI*
	The busy hosts didn't forget people before the party,	so they're probably gonna have enough food to feed the guests.	*negative-bare plural*
	The busy hosts didn't forget nobody before the party,	so they're probably gonna have enough food to feed the guests.	*negative-negative NP*
8	If the stray dog scares anybody with its barking,	then animal control's gonna make it leave the busy neighborhood.	*conditional-NPI*
	If the stray dog scares people with its barking,	then animal control's gonna make it leave the busy neighborhood.	*conditional-bare plural*
	If the stray dog scares nobody with its barking,	then animal control's gonna make it leave the busy neighborhood.	*conditional-negative NP*
	The stray dog didn't scare anybody with its barking,	so animal control's gonna let it stay in the busy neighborhood.	*negative-NPI*
	The stray dog didn't scare people with its barking,	so animal control's gonna let it stay in the busy neighborhood.	*negative-bare plural*
	The stray dog didn't scare nobody with its barking,	so animal control's gonna let it stay in the busy neighborhood.	*negative-negative NP*
9	If the math teacher helps anybody before the test,	then the students are gonna write nice stuff in their evaluations.	*conditional-NPI*
	If the math teacher helps people before the test,	then the students are gonna write nice stuff in their evaluations.	*conditional-bare plural*
	If the math teacher helps nobody before the test,	then the students are gonna write nice stuff in their evaluations.	*conditional-negative NP*
	The math teacher didn't help anybody before the test,	so the students are gonna write mean stuff in their evaluations.	*negative-NPI*
	The math teacher didn't help people before the test,	so the students are gonna write mean stuff in their evaluations.	*negative-bare plural*
	The math teacher didn't help nobody before the test,	so the students are gonna write mean stuff in their evaluations.	*negative-negative NP*
10	If the new ushers help anybody to their seats,	then the manager is gonna compliment them at the next meeting.	*conditional-NPI*
	If the new ushers help people to their seats,	then the manager is gonna compliment them at the next meeting.	*conditional-bare plural*
	If the new ushers help nobody to their seats,	then the manager is gonna compliment them at the next meeting.	*conditional-negative NP*
	The new ushers didn't help anybody to their seats,	so the manager is gonna reprimand them at the next meeting.	*negative-NPI*
	The new ushers didn't help people to their seats,	so the manager is gonna reprimand them at the next meeting.	*negative-bare plural*
	The new ushers didn't help nobody to their seats,	so the manager is gonna reprimand them at the next meeting.	*negative-negative NP*

11	If the football fans hurt anybody during the fight,	then the police are gonna crack down hard on all of them for it.	*conditional-NPI*
	If the football fans hurt people during the fight,	then the police are gonna crack down hard on all of them for it.	*conditional-bare plural*
	If the football fans hurt nobody during the fight,	then the police are gonna crack down hard on all of them for it.	*conditional-negative NP*
	The football fans didn't hurt anybody during the fight,	so the police are gonna go pretty easy on all of them for it.	*negative-NPI*
	The football fans didn't hurt people during the fight,	so the police are gonna go pretty easy on all of them for it.	*negative-bare plural*
	The football fans didn't hurt nobody during the fight,	so the police are gonna go pretty easy on all of them for it.	*negative-negative NP*
12	If our shy roommate invites anybody to the game night,	then he's probably gonna feel comfortable making conversation.	*conditional-NPI*
	If our shy roommate invites people to the game night,	then he's probably gonna feel comfortable making conversation.	*conditional-bare plural*
	If our shy roommate invites nobody to the game night,	then he's probably gonna feel comfortable making conversation.	*conditional-negative NP*
	Our shy roommate didn't invite anybody to the game night,	so he's probably gonna feel awkward making conversation.	*negative-NPI*
	Our shy roommate didn't invite people to the game night,	so he's probably gonna feel awkward making conversation.	*negative-bare plural*
	Our shy roommate didn't invite nobody to the game night,	so he's probably gonna feel awkward making conversation.	*negative-negative NP*
13	If the soccer coach praises anybody during tryouts,	then the players are gonna be pretty sure who'll be on the team.	*conditional-NPI*
	If the soccer coach praises people during tryouts,	then the players are gonna be pretty sure who'll be on the team.	*conditional-bare plural*
	If the soccer coach praises nobody during tryouts,	then the players are gonna be pretty sure who'll be on the team.	*conditional-negative NP*
	The soccer coach didn't praise anybody during tryouts,	so the players are really gonna wonder who'll be on the team.	*negative-NPI*
	The soccer coach didn't praise people during tryouts,	so the players are really gonna wonder who'll be on the team.	*negative-bare plural*
	The soccer coach didn't praise nobody during tryouts,	so the players are really gonna wonder who'll be on the team.	*negative-negative NP*
14	If my big brother meets anybody during training,	then he's probably gonna have company for lunch this week.	*conditional-NPI*
	If my big brother meets people during training,	then he's probably gonna have company for lunch this week.	*conditional-bare plural*
	If my big brother meets nobody during training,	then he's probably gonna have company for lunch this week.	*conditional-negative NP*
	My big brother didn't meet anybody during training,	so he's probably gonna eat by himself for lunch this week.	*negative-NPI*
	My big brother didn't meet people during training,	so he's probably gonna eat by himself for lunch this week.	*negative-bare plural*

	My big brother didn't meet nobody during training,	so he's probably gonna eat by himself for lunch this week.	*negative-negative NP*
15	If the young couple meets anybody before the cruise,	then the vacation is gonna be pretty fun while they're at sea.	*conditional-NPI*
	If the young couple meets people before the cruise,	then the vacation is gonna be pretty fun while they're at sea.	*conditional-bare plural*
	If the young couple meets nobody before the cruise,	then the vacation is gonna be pretty fun while they're at sea.	*conditional-negative NP*
	The young couple didn't meet anybody before the cruise,	so the vacation is gonna be kinda lonely while they're at sea.	*negative-NPI*
	The young couple didn't meet people before the cruise,	so the vacation is gonna be kinda lonely while they're at sea.	*negative-bare plural*
	The young couple didn't meet nobody before the cruise,	so the vacation is gonna be kinda lonely while they're at sea.	*negative-negative NP*
16	If the little kid meets anybody at the playground,	then for a while he's gonna share his toys in the big sandbox.	*conditional-NPI*
	If the little kid meets people at the playground,	then for a while he's gonna share his toys in the big sandbox.	*conditional-bare plural*
	If the little kid meets nobody at the playground,	then for a while he's gonna share his toys in the big sandbox.	*conditional-negative NP*
	The little kid didn't meet anybody at the playground,	so for a while he's gonna play by himself in the big sandbox.	*negative-NPI*
	The little kid didn't meet people at the playground,	so for a while he's gonna play by himself in the big sandbox.	*negative-bare plural*
	The little kid didn't meet nobody at the playground,	so for a while he's gonna play by himself in the big sandbox.	*negative-negative NP*
17	If the head coach watches anybody during practice,	then it's definitely gonna be hard to miss the big improvements.	*conditional-NPI*
	If the head coach watches people during practice,	then it's definitely gonna be hard to miss the big improvements.	*conditional-bare plural*
	If the head coach watches nobody during practice,	then it's definitely gonna be hard to miss the big improvements.	*conditional-negative NP*
	The head coach didn't watch anybody during practice,	so it's definitely gonna be easy to miss the big improvements.	*negative-NPI*
	The head coach didn't watch people during practice,	so it's definitely gonna be easy to miss the big improvements.	*negative-bare plural*
	The head coach didn't watch nobody during practice,	so it's definitely gonna be easy to miss the big improvements.	*negative-negative NP*
18	If the big puppy scares anybody during her walk,	then the owner is gonna scold her for it when they get home.	*conditional-NPI*
	If the big puppy scares people during her walk,	then the owner is gonna scold her for it when they get home.	*conditional-bare plural*
	If the big puppy scares nobody during her walk,	then the owner is gonna scold her for it when they get home.	*conditional-negative NP*
	The big puppy didn't scare anybody during her walk,	so the owner is gonna give her a treat when they get home.	*negative-NPI*

	The big puppy didn't scare people during her walk,	so the owner is gonna give her a treat when they get home.	*negative-bare plural*
	The big puppy didn't scare nobody during her walk,	so the owner is gonna give her a treat when they get home.	*negative-negative NP*
19	If the hairy spider scares anybody from its corner,	then my roommates are gonna leave the room for a little while.	*conditional-NPI*
	If the hairy spider scares people from its corner,	then my roommates are gonna leave the room for a little while.	*conditional-bare plural*
	If the hairy spider scares nobody from its corner,	then my roommates are gonna leave the room for a little while.	*conditional-negative NP*
	The hairy spider didn't scare anybody from its corner,	so my roommates are gonna let it hang out for a little while.	*negative-NPI*
	The hairy spider didn't scare people from its corner,	so my roommates are gonna let it hang out for a little while.	*negative-bare plural*
	The hairy spider didn't scare nobody from its corner,	so my roommates are gonna let it hang out for a little while.	*negative-negative NP*
20	If the star pitcher tells anybody about her injury,	then her coach is gonna understand why she's playing so badly.	*conditional-NPI*
	If the star pitcher tells people about her injury,	then her coach is gonna understand why she's playing so badly.	*conditional-bare plural*
	If the star pitcher tells nobody about her injury,	then her coach is gonna understand why she's playing so badly.	*conditional-negative NP*
	The star pitcher didn't tell anybody about her injury,	so her coach is gonna be confused that she's playing so badly.	*negative-NPI*
	The star pitcher didn't tell people about her injury,	so her coach is gonna be confused that she's playing so badly.	*negative-bare plural*
	The star pitcher didn't tell nobody about her injury,	so her coach is gonna be confused that she's playing so badly.	*negative-negative NP*
21	If the hair stylist tells anybody about his trip,	then his regulars are gonna understand where he was all last month.	*conditional-NPI*
	If the hair stylist tells people about his trip,	then his regulars are gonna understand where he was all last month.	*conditional-bare plural*
	If the hair stylist tells nobody about his trip,	then his regulars are gonna understand where he was all last month.	*conditional-negative NP*
	The hair stylist didn't tell anybody about his trip,	so his regulars are gonna wonder where he was all last month.	*negative-NPI*
	The hair stylist didn't tell people about his trip,	so his regulars are gonna wonder where he was all last month.	*negative-bare plural*
	The hair stylist didn't tell nobody about his trip,	so his regulars are gonna wonder where he was all last month.	*negative-negative NP*
22	If the band members tell anybody about their show,	then the crowd is gonna be a good size for this new venue.	*conditional-NPI*

	If the band members tell people about their show,	then the crowd is gonna be a good size for this new venue.	*conditional-bare plural*
	If the band members tell nobody about their show,	then the crowd is gonna be a good size for this new venue.	*conditional-negative NP*
	The band members didn't tell anybody about their show,	so the crowd is gonna be pretty small for this new venue.	*negative-NPI*
	The band members didn't tell people about their show,	so the crowd is gonna be pretty small for this new venue.	*negative-bare plural*
	The band members didn't tell nobody about their show,	so the crowd is gonna be pretty small for this new venue.	*negative-negative NP*
23	If the night nurse tells anybody about jury duty,	then the scheduler is gonna plan way ahead to cover her shifts.	*conditional-NPI*
	If the night nurse tells people about jury duty,	then the scheduler is gonna plan way ahead to cover her shifts.	*conditional-bare plural*
	If the night nurse tells nobody about jury duty,	then the scheduler is gonna plan way ahead to cover her shifts.	*conditional-negative NP*
	The night nurse didn't tell anybody about jury duty,	so the scheduler is gonna have to scramble to cover her shifts.	*negative-NPI*
	The night nurse didn't tell people about jury duty,	so the scheduler is gonna have to scramble to cover her shifts.	*negative-bare plural*
	The night nurse didn't tell nobody about jury duty,	so the scheduler is gonna have to scramble to cover her shifts.	*negative-negative NP*
24	If the news anchor warns anybody about the floods,	then most folks are gonna know it's risky to stay in their homes.	*conditional-NPI*
	If the news anchor warns people about the floods,	then most folks are gonna know it's risky to stay in their homes.	*conditional-bare plural*
	If the news anchor warns nobody about the floods,	then most folks are gonna know it's risky to stay in their homes.	*conditional-negative NP*
	The news anchor didn't warn anybody about the floods,	so most folks are gonna think it's safe to stay in their homes.	*negative-NPI*
	The news anchor didn't warn people about the floods,	so most folks are gonna think it's safe to stay in their homes.	*negative-bare plural*
	The news anchor didn't warn nobody about the floods,	so most folks are gonna think it's safe to stay in their homes.	*negative-negative NP*
25	If the mail carrier brings anything Saturday morning,	then the present is gonna get there before the birthday party.	*conditional-NPI*
	If the mail carrier brings things Saturday morning,	then the present is gonna get there before the birthday party.	*conditional-bare plural*
	If the mail carrier brings nothing Saturday morning,	then the present is gonna get there before the birthday party.	*conditional-negative NP*
	The mail carrier didn't bring anything Saturday morning,	so the present is gonna get there after the birthday party.	*negative-NPI*
	The mail carrier didn't bring things Saturday morning,	so the present is gonna get there after the birthday party.	*negative-bare plural*
	The mail carrier didn't bring nothing Saturday morning,	so the present is gonna get there after the birthday party.	*negative-negative NP*

26	If the airline pilot checks anything ahead of time,	then it's probably gonna be a short wait before they take off.	*conditional-NPI*
	If the airline pilot checks things ahead of time,	then it's probably gonna be a short wait before they take off.	*conditional-bare plural*
	If the airline pilot checks nothing ahead of time,	then it's probably gonna be a short wait before they take off.	*conditional-negative NP*
	The airline pilot didn't check anything ahead of time,	so it's probably gonna be a long wait before they take off.	*negative-NPI*
	The airline pilot didn't check things ahead of time,	so it's probably gonna be a long wait before they take off.	*negative-bare plural*
	The airline pilot didn't check nothing ahead of time,	so it's probably gonna be a long wait before they take off.	*negative-negative NP*
27	If the tech worker codes anything during her shift,	then the managers are gonna be sure that she was doing her job.	*conditional-NPI*
	If the tech worker codes things during her shift,	then the managers are gonna be sure that she was doing her job.	*conditional-bare plural*
	If the tech worker codes nothing during her shift,	then the managers are gonna be sure that she was doing her job.	*conditional-negative NP*
	The tech worker didn't code anything during her shift,	so the managers are gonna wonder whether she was doing her job.	*negative-NPI*
	The tech worker didn't code things during her shift,	so the managers are gonna wonder whether she was doing her job.	*negative-bare plural*
	The tech worker didn't code nothing during her shift,	so the managers are gonna wonder whether she was doing her job.	*negative-negative NP*
28	If the art student draws anything on her canvas,	then her teacher is gonna be sure that she's paying attention.	*conditional-NPI*
	If the art student draws things on her canvas,	then her teacher is gonna be sure that she's paying attention.	*conditional-bare plural*
	If the art student draws nothing on her canvas,	then her teacher is gonna be sure that she's paying attention.	*conditional-negative NP*
	The art student didn't draw anything on her canvas,	so her teacher is gonna wonder whether she's paying attention.	*negative-NPI*
	The art student didn't draw things on her canvas,	so her teacher is gonna wonder whether she's paying attention.	*negative-bare plural*
	The art student didn't draw nothing on her canvas,	so her teacher is gonna wonder whether she's paying attention.	*negative-negative NP*
29	If the office worker files anything in the cabinet,	then her new boss is gonna be satisfied when she gets in today.	*conditional-NPI*
	If the office worker files things in the cabinet,	then her new boss is gonna be satisfied when she gets in today.	*conditional-bare plural*
	If the office worker files nothing in the cabinet,	then her new boss is gonna be satisfied when she gets in today.	*conditional-negative NP*
	The office worker didn't file anything in the cabinet,	so her new boss is gonna be irritated when she gets in today.	*negative-NPI*
	The office worker didn't file things in the cabinet,	so her new boss is gonna be irritated when she gets in today.	*negative-bare plural*

	The office worker didn't file nothing in the cabinet,	so her new boss is gonna be irritated when she gets in today.	*negative-negative NP*
30	If the black cat finds anything in her food dish,	then she's probably gonna eat her food and then take a nap.	*conditional-NPI*
	If the black cat finds things in her food dish,	then she's probably gonna eat her food and then take a nap.	*conditional-bare plural*
	If the black cat finds nothing in her food dish,	then she's probably gonna eat her food and then take a nap.	*conditional-negative NP*
	The black cat didn't find anything in her food dish,	so she's probably gonna catch a mouse and then take a nap.	*negative-NPI*
	The black cat didn't find things in her food dish,	so she's probably gonna catch a mouse and then take a nap.	*negative-bare plural*
	The black cat didn't find nothing in her food dish,	so she's probably gonna catch a mouse and then take a nap.	*negative-negative NP*
31	If the new teacher hangs anything in the classroom,	then the walls are gonna be more colorful when the kids show up.	*conditional-NPI*
	If the new teacher hangs things in the classroom,	then the walls are gonna be more colorful when the kids show up.	*conditional-bare plural*
	If the new teacher hangs nothing in the classroom,	then the walls are gonna be more colorful when the kids show up.	*conditional-negative NP*
	The new teacher didn't hang anything in the classroom,	so the walls are gonna be pretty boring when the kids show up.	*negative-NPI*
	The new teacher didn't hang things in the classroom,	so the walls are gonna be pretty boring when the kids show up.	*negative-bare plural*
	The new teacher didn't hang nothing in the classroom,	so the walls are gonna be pretty boring when the kids show up.	*negative-negative NP*
32	If the sleepy kid hears anything after lights-out,	then the dark room is gonna seem way scarier than it usually does.	*conditional-NPI*
	If the sleepy kid hears things after lights-out,	then the dark room is gonna seem way scarier than it usually does.	*conditional-bare plural*
	If the sleepy kid hears nothing after lights-out,	then the dark room is gonna seem way scarier than it usually does.	*conditional-negative NP*
	The sleepy kid didn't hear anything after lights-out,	so the dark room is gonna seem less scary than it usually does.	*negative-NPI*
	The sleepy kid didn't hear things after lights-out,	so the dark room is gonna seem less scary than it usually does.	*negative-bare plural*
	The sleepy kid didn't hear nothing after lights-out,	so the dark room is gonna seem less scary than it usually does.	*negative-negative NP*
33	If the old screens keep anything out of the house,	then the landlord is gonna leave them there once it gets warmer.	*conditional-NPI*
	If the old screens keep things out of the house,	then the landlord is gonna leave them there once it gets warmer.	*conditional-bare plural*
	If the old screens keep nothing out of the house,	then the landlord is gonna leave them there once it gets warmer.	*conditional-negative NP*
	The old screens didn't keep anything out of the house,	so the landlord is gonna put new ones in once it gets warmer.	*negative-NPI*

	The old screens didn't keep things out of the house,	so the landlord is gonna put new ones in once it gets warmer.	*negative-bare plural*
	The old screens didn't keep nothing out of the house,	so the landlord is gonna put new ones in once it gets warmer.	*negative-negative NP*
34	If my older sister leaves anything in her locker,	then her backpack is gonna be a bit lighter during her walk home.	*conditional-NPI*
	If my older sister leaves things in her locker,	then her backpack is gonna be a bit lighter during her walk home.	*conditional-bare plural*
	If my older sister leaves nothing in her locker,	then her backpack is gonna be a bit lighter during her walk home.	*conditional-negative NP*
	My older sister didn't leave anything in her locker,	so her backpack is gonna be super heavy during her walk home.	*negative-NPI*
	My older sister didn't leave things in her locker,	so her backpack is gonna be super heavy during her walk home.	*negative-bare plural*
	My older sister didn't leave nothing in her locker,	so her backpack is gonna be super heavy during her walk home.	*negative-negative NP*
35	If the bank teller orders anything over the weekend,	then her bills are gonna be kinda high for this last month.	*conditional-NPI*
	If the bank teller orders things over the weekend,	then her bills are gonna be kinda high for this last month.	*conditional-bare plural*
	If the bank teller orders nothing over the weekend,	then her bills are gonna be kinda high for this last month.	*conditional-negative NP*
	The bank teller didn't order anything over the weekend,	so her bills are gonna be pretty low for this last month.	*negative-NPI*
	The bank teller didn't order things over the weekend,	so her bills are gonna be pretty low for this last month.	*negative-bare plural*
	The bank teller didn't order nothing over the weekend,	so her bills are gonna be pretty low for this last month.	*negative-negative NP*
36	If the bus driver packs anything for lunch today,	then she's probably gonna eat on the bus later this afternoon.	*conditional-NPI*
	If the bus driver packs things for lunch today,	then she's probably gonna eat on the bus later this afternoon.	*conditional-bare plural*
	If the bus driver packs nothing for lunch today,	then she's probably gonna eat on the bus later this afternoon.	*conditional-negative NP*
	The bus driver didn't pack anything for lunch today,	so she's probably gonna stop for food later this afternoon.	*negative-NPI*
	The bus driver didn't pack things for lunch today,	so she's probably gonna stop for food later this afternoon.	*negative-bare plural*
	The bus driver didn't pack nothing for lunch today,	so she's probably gonna stop for food later this afternoon.	*negative-negative NP*
37	If my little cousin plans anything for Labor Day,	then the family's gonna let her miss the annual barbecue.	*conditional-NPI*
	If my little cousin plans things for Labor Day,	then the family's gonna let her miss the annual barbecue.	*conditional-bare plural*
	If my little cousin plans nothing for Labor Day,	then the family's gonna let her miss the annual barbecue.	*conditional-negative NP*

	My little cousin didn't plan anything for Labor Day,	so the family's gonna make her come to the annual barbecue.	*negative-NPI*
	My little cousin didn't plan things for Labor Day,	so the family's gonna make her come to the annual barbecue.	*negative-bare plural*
	My little cousin didn't plan nothing for Labor Day,	so the family's gonna make her come to the annual barbecue.	*negative-negative NP*
38	If my grandfather plants anything in his garden,	then my grandma is gonna go to the garden for fresh vegetables.	*conditional-NPI*
	If my grandfather plants things in his garden,	then my grandma is gonna go to the garden for fresh vegetables.	*conditional-bare plural*
	If my grandfather plants nothing in his garden,	then my grandma is gonna go to the garden for fresh vegetables.	*conditional-negative NP*
	My grandfather didn't plant anything in his garden,	so my grandma is gonna go to the store for fresh vegetables.	*negative-NPI*
	My grandfather didn't plant things in his garden,	so my grandma is gonna go to the store for fresh vegetables.	*negative-bare plural*
	My grandfather didn't plant nothing in his garden,	so my grandma is gonna go to the store for fresh vegetables.	*negative-negative NP*
39	If the head cook preps anything in the afternoon,	then the wait time is gonna be pretty short during the dinner rush.	*conditional-NPI*
	If the head cook preps things in the afternoon,	then the wait time is gonna be pretty short during the dinner rush.	*conditional-bare plural*
	If the head cook preps nothing in the afternoon,	then the wait time is gonna be pretty short during the dinner rush.	*conditional-negative NP*
	The head cook didn't prep anything in the afternoon,	so the wait time is gonna be really long during the dinner rush.	*negative-NPI*
	The head cook didn't prep things in the afternoon,	so the wait time is gonna be really long during the dinner rush.	*negative-bare plural*
	The head cook didn't prep nothing in the afternoon,	so the wait time is gonna be really long during the dinner rush.	*negative-negative NP*
40	If the lazy student reads anything over spring break,	then she's probably gonna be all caught up when she gets back.	*conditional-NPI*
	If the lazy student reads things over spring break,	then she's probably gonna be all caught up when she gets back.	*conditional-bare plural*
	If the lazy student reads nothing over spring break,	then she's probably gonna be all caught up when she gets back.	*conditional-negative NP*
	The lazy student didn't read anything over spring break,	so she's probably gonna have to catch up when she gets back.	*negative-NPI*
	The lazy student didn't read things over spring break,	so she's probably gonna have to catch up when she gets back.	*negative-bare plural*
	The lazy student didn't read nothing over spring break,	so she's probably gonna have to catch up when she gets back.	*negative-negative NP*
41	If the young kid yells anything during the play,	then the family is gonna feel bad about staying to the end.	*conditional-NPI*
	If the young kid yells things during the play,	then the family is gonna feel bad about staying to the end.	*conditional-bare plural*

	If the young kid yells nothing during the play,	then the family is gonna feel bad about staying to the end.	*conditional-negative NP*
	The young kid didn't yell anything during the play,	so the family is gonna feel okay about staying to the end.	*negative-NPI*
	The young kid didn't yell things during the play,	so the family is gonna feel okay about staying to the end.	*negative-bare plural*
	The young kid didn't yell nothing during the play,	so the family is gonna feel okay about staying to the end.	*negative-negative NP*
42	If the sixth-grader recalls anything during the test,	then his grade is gonna be pretty okay compared to other kids.	*conditional-NPI*
	If the sixth-grader recalls things during the test,	then his grade is gonna be pretty okay compared to other kids.	*conditional-bare plural*
	If the sixth-grader recalls nothing during the test,	then his grade is gonna be pretty okay compared to other kids.	*conditional-negative NP*
	The sixth-grader didn't recall anything during the test,	so his grade is gonna be really bad compared to other kids.	*negative-NPI*
	The sixth-grader didn't recall things during the test,	so his grade is gonna be really bad compared to other kids.	*negative-bare plural*
	The sixth-grader didn't recall nothing during the test,	so his grade is gonna be really bad compared to other kids.	*negative-negative NP*
43	If the ski store sells anything to the locals,	then it's probably gonna stay in business for the off-season.	*conditional-NPI*
	If the ski store sells things to the locals,	then it's probably gonna stay in business for the off-season.	*conditional-bare plural*
	If the ski store sells nothing to the locals,	then it's probably gonna stay in business for the off-season.	*conditional-negative NP*
	The ski store didn't sell anything to the locals,	so it's probably gonna end up closing for the off-season.	*negative-NPI*
	The ski store didn't sell things to the locals,	so it's probably gonna end up closing for the off-season.	*negative-bare plural*
	The ski store didn't sell nothing to the locals,	so it's probably gonna end up closing for the off-season.	*negative-negative NP*
44	If the hotdog stand sells anything during the storm,	then the owners are gonna keep it open next time it snows.	*conditional-NPI*
	If the hotdog stand sells things during the storm,	then the owners are gonna keep it open next time it snows.	*conditional-bare plural*
	If the hotdog stand sells nothing during the storm,	then the owners are gonna keep it open next time it snows.	*conditional-negative NP*
	The hotdog stand didn't sell anything during the storm,	so the owners are gonna close it down next time it snows.	*negative-NPI*
	The hotdog stand didn't sell things during the storm,	so the owners are gonna close it down next time it snows.	*negative-bare plural*
	The hotdog stand didn't sell nothing during the storm,	so the owners are gonna close it down next time it snows.	*negative-negative NP*
45	If my best friend studies anything during the review,	then she's probably gonna do pretty well on the exam tomorrow.	*conditional-NPI*

	If my best friend studies things during the review,	then she's probably gonna do pretty well on the exam tomorrow.	*conditional-bare plural*
	If my best friend studies nothing during the review,	then she's probably gonna do pretty well on the exam tomorrow.	*conditional-negative NP*
	My best friend didn't study anything during the review,	so she's probably gonna do really bad on the exam tomorrow.	*negative-NPI*
	My best friend didn't study things during the review,	so she's probably gonna do really bad on the exam tomorrow.	*negative-bare plural*
	My best friend didn't study nothing during the review,	so she's probably gonna do really bad on the exam tomorrow.	*negative-negative NP*
46	If the little boy takes anything for lunch today,	then his lunchbox is gonna have food in it when it's time to eat.	*conditional-NPI*
	If the little boy takes things for lunch today,	then his lunchbox is gonna have food in it when it's time to eat.	*conditional-bare plural*
	If the little boy takes nothing for lunch today,	then his lunchbox is gonna have food in it when it's time to eat.	*conditional-negative NP*
	The little boy didn't take anything for lunch today,	so his lunchbox is gonna be totally empty when it's time to eat.	*negative-NPI*
	The little boy didn't take things for lunch today,	so his lunchbox is gonna be totally empty when it's time to eat.	*negative-bare plural*
	The little boy didn't take nothing for lunch today,	so his lunchbox is gonna be totally empty when it's time to eat.	*negative-negative NP*
47	If the guest speaker plans anything before her speech,	then she is probably gonna sound convincing during the lecture.	*conditional-NPI*
	If the guest speaker plans things before her speech,	then she is probably gonna sound convincing during the lecture.	*conditional-bare plural*
	If the guest speaker plans nothing before her speech,	then she is probably gonna sound convincing during the lecture.	*conditional-negative NP*
	The guest speaker didn't plan anything before her speech,	so she is probably gonna sound flustered during the lecture.	*negative-NPI*
	The guest speaker didn't plan things before her speech,	so she is probably gonna sound flustered during the lecture.	*negative-bare plural*
	The guest speaker didn't plan nothing before her speech,	so she is probably gonna sound flustered during the lecture.	*negative-negative NP*
48	If the famous author writes anything over the summer,	then her readers are gonna get her new book to read before long.	*conditional-NPI*
	If the famous author writes things over the summer,	then her readers are gonna get her new book to read before long.	*conditional-bare plural*
	If the famous author writes nothing over the summer,	then her readers are gonna get her new book to read before long.	*conditional-negative NP*
	The famous author didn't write anything over the summer,	so her readers are gonna be out of books to read before long.	*negative-NPI*
	The famous author didn't write things over the summer,	so her readers are gonna be out of books to read before long.	*negative-bare plural*
	The famous author didn't write nothing over the summer,	so her readers are gonna be out of books to read before long.	*negative-negative NP*

Table A.2 Critical sentences from the follow-up survey. Each participant saw only one version of each item, in a Latin Square design. This resulted in 3 lists.

Item	First Clause	Continuation	Trial Type
1	If the loud music bothers anybody during dinner,	then the friends are gonna stay for a drink after they've eaten.	*conditional-NPI*
	If the loud music bothers people during dinner,	then the friends are gonna stay for a drink after they've eaten.	*conditional-bare plural*
	If the loud music bothers nobody during dinner,	then the friends are gonna stay for a drink after they've eaten.	*conditional-negative NP*
2	If my favorite aunt brings anybody to the cookout,	then I bet she is gonna end up bored the entire evening.	*conditional-NPI*
	If my favorite aunt brings people to the cookout,	then I bet she is gonna end up bored the entire evening.	*conditional-bare plural*
	If my favorite aunt brings nobody to the cookout,	then I bet she is gonna end up bored the entire evening.	*conditional-negative NP*
3	If the store manager fires anybody for late arrival,	then the workers are gonna get careless about being prompt.	*conditional-NPI*
	If the store manager fires people for late arrival,	then the workers are gonna get careless about being prompt.	*conditional-bare plural*
	If the store manager fires nobody for late arrival,	then the workers are gonna get careless about being prompt.	*conditional-negative NP*
4	If the excited kid follows anybody from her group,	then she is probably gonna lose the others in the science museum.	*conditional-NPI*
	If the excited kid follows people from her group,	then she is probably gonna lose the others in the science museum.	*conditional-bare plural*
	If the excited kid follows nobody from her group,	then she is probably gonna lose the others in the science museum.	*conditional-negative NP*
5	If the piano student follows anybody at the rehearsal,	then she is probably gonna be really lost during the performance.	*conditional-NPI*
	If the piano student follows people at the rehearsal,	then she is probably gonna be really lost during the performance.	*conditional-bare plural*
	If the piano student follows nobody at the rehearsal,	then she is probably gonna be really lost during the performance.	*conditional-negative NP*
6	If the six-year-old fools anybody with her story,	then her family is gonna know that she broke the dinner plate.	*conditional-NPI*
	If the six-year-old fools people with her story,	then her family is gonna know that she broke the dinner plate.	*conditional-bare plural*
	If the six-year-old fools nobody with her story,	then her family is gonna know that she broke the dinner plate.	*conditional-negative NP*
7	If the busy hosts forget anybody before the party,	then they're probably gonna have enough food to feed the guests.	*conditional-NPI*
	If the busy hosts forget people before the party,	then they're probably gonna have enough food to feed the guests.	*conditional-bare plural*

	If the busy hosts forget nobody before the party,	then they're probably gonna have enough food to feed the guests.	*conditional-negative NP*
8	If the stray dog scares anybody with its barking,	then animal control's gonna let it stay in the busy neighborhood.	*conditional-NPI*
	If the stray dog scares people with its barking,	then animal control's gonna let it stay in the busy neighborhood.	*conditional-bare plural*
	If the stray dog scares nobody with its barking,	then animal control's gonna let it stay in the busy neighborhood.	*conditional-negative NP*
9	If the math teacher helps anybody before the test,	then the students are gonna write mean stuff in their evaluations.	*conditional-NPI*
	If the math teacher helps people before the test,	then the students are gonna write mean stuff in their evaluations.	*conditional-bare plural*
	If the math teacher helps nobody before the test,	then the students are gonna write mean stuff in their evaluations.	*conditional-negative NP*
10	If the new ushers help anybody to their seats,	then the manager is gonna reprimand them at the next meeting.	*conditional-NPI*
	If the new ushers help people to their seats,	then the manager is gonna reprimand them at the next meeting.	*conditional-bare plural*
	If the new ushers help nobody to their seats,	then the manager is gonna reprimand them at the next meeting.	*conditional-negative NP*
11	If the football fans hurt anybody during the fight,	then the police are gonna go pretty easy on all of them for it.	*conditional-NPI*
	If the football fans hurt people during the fight,	then the police are gonna go pretty easy on all of them for it.	*conditional-bare plural*
	If the football fans hurt nobody during the fight,	then the police are gonna go pretty easy on all of them for it.	*conditional-negative NP*
12	If our shy roommate invites anybody to the game night,	then he's probably gonna feel awkward making conversation.	*conditional-NPI*
	If our shy roommate invites people to the game night,	then he's probably gonna feel awkward making conversation.	*conditional-bare plural*
	If our shy roommate invites nobody to the game night,	then he's probably gonna feel awkward making conversation.	*conditional-negative NP*
13	If the soccer coach praises anybody during tryouts,	then the players are really gonna wonder who'll be on the team.	*conditional-NPI*
	If the soccer coach praises people during tryouts,	then the players are really gonna wonder who'll be on the team.	*conditional-bare plural*
	If the soccer coach praises nobody during tryouts,	then the players are really gonna wonder who'll be on the team.	*conditional-negative NP*
14	If my big brother meets anybody during training,	then he's probably gonna eat by himself for lunch this week.	*conditional-NPI*
	If my big brother meets people during training,	then he's probably gonna eat by himself for lunch this week.	*conditional-bare plural*

	If my big brother meets nobody during training,	then he's probably gonna eat by himself for lunch this week.	*conditional-negative NP*
15	If the young couple meets anybody before the cruise,	then the vacation is gonna be kinda lonely while they're at sea.	*conditional-NPI*
	If the young couple meets people before the cruise,	then the vacation is gonna be kinda lonely while they're at sea.	*conditional-bare plural*
	If the young couple meets nobody before the cruise,	then the vacation is gonna be kinda lonely while they're at sea.	*conditional-negative NP*
16	If the little kid meets anybody at the playground,	then for a while he's gonna play by himself in the big sandbox.	*conditional-NPI*
	If the little kid meets people at the playground,	then for a while he's gonna play by himself in the big sandbox.	*conditional-bare plural*
	If the little kid meets nobody at the playground,	then for a while he's gonna play by himself in the big sandbox.	*conditional-negative NP*
17	If the head coach watches anybody during practice,	then it's definitely gonna be easy to miss the big improvements.	*conditional-NPI*
	If the head coach watches people during practice,	then it's definitely gonna be easy to miss the big improvements.	*conditional-bare plural*
	If the head coach watches nobody during practice,	then it's definitely gonna be easy to miss the big improvements.	*conditional-negative NP*
18	If the big puppy scares anybody during her walk,	then the owner is gonna give her a treat when they get home.	*conditional-NPI*
	If the big puppy scares people during her walk,	then the owner is gonna give her a treat when they get home.	*conditional-bare plural*
	If the big puppy scares nobody during her walk,	then the owner is gonna give her a treat when they get home.	*conditional-negative NP*
19	If the hairy spider scares anybody from its corner,	then my roommates are gonna let it hang out for a little while.	*conditional-NPI*
	If the hairy spider scares people from its corner,	then my roommates are gonna let it hang out for a little while.	*conditional-bare plural*
	If the hairy spider scares nobody from its corner,	then my roommates are gonna let it hang out for a little while.	*conditional-negative NP*
20	If the star pitcher tells anybody about her injury,	then her coach is gonna be confused that she's playing so badly.	*conditional-NPI*
	If the star pitcher tells people about her injury,	then her coach is gonna be confused that she's playing so badly.	*conditional-bare plural*
	If the star pitcher tells nobody about her injury,	then her coach is gonna be confused that she's playing so badly.	*conditional-negative NP*
21	If the hair stylist tells anybody about his trip,	then his regulars are gonna wonder where he was all last month.	*conditional-NPI*
	If the hair stylist tells people about his trip,	then his regulars are gonna wonder where he was all last month.	*conditional-bare plural*

	If the hair stylist tells nobody about his trip,	then his regulars are gonna wonder where he was all last month.	*conditional-negative NP*
22	If the band members tell anybody about their show,	then the crowd is gonna be pretty small for this new venue.	*conditional-NPI*
	If the band members tell people about their show,	then the crowd is gonna be pretty small for this new venue.	*conditional-bare plural*
	If the band members tell nobody about their show,	then the crowd is gonna be pretty small for this new venue.	*conditional-negative NP*
23	If the night nurse tells anybody about jury duty,	then the scheduler is gonna have to scramble to cover her shifts.	*conditional-NPI*
	If the night nurse tells people about jury duty,	then the scheduler is gonna have to scramble to cover her shifts.	*conditional-bare plural*
	If the night nurse tells nobody about jury duty,	then the scheduler is gonna have to scramble to cover her shifts.	*conditional-negative NP*
24	If the news anchor warns anybody about the floods,	then most folks are gonna think it's safe to stay in their homes.	*conditional-NPI*
	If the news anchor warns people about the floods,	then most folks are gonna think it's safe to stay in their homes.	*conditional-bare plural*
	If the news anchor warns nobody about the floods,	then most folks are gonna think it's safe to stay in their homes.	*conditional-negative NP*
25	If the mail carrier brings anything Saturday morning,	then the present is gonna get there after the birthday party.	*conditional-NPI*
	If the mail carrier brings things Saturday morning,	then the present is gonna get there after the birthday party.	*conditional-bare plural*
	If the mail carrier brings nothing Saturday morning,	then the present is gonna get there after the birthday party.	*conditional-negative NP*
26	If the airline pilot checks anything ahead of time,	then it's probably gonna be a long wait before they take off.	*conditional-NPI*
	If the airline pilot checks things ahead of time,	then it's probably gonna be a long wait before they take off.	*conditional-bare plural*
	If the airline pilot checks nothing ahead of time,	then it's probably gonna be a long wait before they take off.	*conditional-negative NP*
27	If the tech worker codes anything during her shift,	then the managers are gonna wonder whether she was doing her job.	*conditional-NPI*
	If the tech worker codes things during her shift,	then the managers are gonna wonder whether she was doing her job.	*conditional-bare plural*
	If the tech worker codes nothing during her shift,	then the managers are gonna wonder whether she was doing her job.	*conditional-negative NP*
28	If the art student draws anything on her canvas,	then her teacher is gonna wonder whether she's paying attention.	*conditional-NPI*
	If the art student draws things on her canvas,	then her teacher is gonna wonder whether she's paying attention.	*conditional-bare plural*

	If the art student draws nothing on her canvas,	then her teacher is gonna wonder whether she's paying attention.	*conditional-negative NP*
29	If the office worker files anything in the cabinet,	then her new boss is gonna be irritated when she gets in today.	*conditional-NPI*
	If the office worker files things in the cabinet,	then her new boss is gonna be irritated when she gets in today.	*conditional-bare plural*
	If the office worker files nothing in the cabinet,	then her new boss is gonna be irritated when she gets in today.	*conditional-negative NP*
30	If the black cat finds anything in her food dish,	then she's probably gonna catch a mouse and then take a nap.	*conditional-NPI*
	If the black cat finds things in her food dish,	then she's probably gonna catch a mouse and then take a nap.	*conditional-bare plural*
	If the black cat finds nothing in her food dish,	then she's probably gonna catch a mouse and then take a nap.	*conditional-negative NP*
31	If the new teacher hangs anything in the classroom,	then the walls are gonna be pretty boring when the kids show up.	*conditional-NPI*
	If the new teacher hangs things in the classroom,	then the walls are gonna be pretty boring when the kids show up.	*conditional-bare plural*
	If the new teacher hangs nothing in the classroom,	then the walls are gonna be pretty boring when the kids show up.	*conditional-negative NP*
32	If the sleepy kid hears anything after lights-out,	then the dark room is gonna seem less scary than it usually does.	*conditional-NPI*
	If the sleepy kid hears things after lights-out,	then the dark room is gonna seem less scary than it usually does.	*conditional-bare plural*
	If the sleepy kid hears nothing after lights-out,	then the dark room is gonna seem less scary than it usually does.	*conditional-negative NP*
33	If the old screens keep anything out of the house,	then the lazy owner's gonna put new ones in once it gets warmer.	*conditional-NPI*
	If the old screens keep things out of the house,	then the lazy owner's gonna put new ones in once it gets warmer.	*conditional-bare plural*
	If the old screens keep nothing out of the house,	then the lazy owner's gonna put new ones in once it gets warmer.	*conditional-negative NP*
34	If my older sister leaves anything in her locker,	then her backpack is gonna be super heavy during her walk home.	*conditional-NPI*
	If my older sister leaves things in her locker,	then her backpack is gonna be super heavy during her walk home.	*conditional-bare plural*
	If my older sister leaves nothing in her locker,	then her backpack is gonna be super heavy during her walk home.	*conditional-negative NP*
35	If the bank teller orders anything over the weekend,	then her bills are gonna be pretty low for this last month.	*conditional-NPI*
	If the bank teller orders things over the weekend,	then her bills are gonna be pretty low for this last month.	*conditional-bare plural*
	If the bank teller orders nothing over the weekend,	then her bills are gonna be pretty low for this last month.	*conditional-negative NP*
36	If the bus driver packs anything for lunch today,	then she's probably gonna stop for food later this afternoon.	*conditional-NPI*

	If the bus driver packs things for lunch today,	then she's probably gonna stop for food later this afternoon.	*conditional-bare plural*
	If the bus driver packs nothing for lunch today,	then she's probably gonna stop for food later this afternoon.	*conditional-negative NP*
37	If my little cousin plans anything for Labor Day,	then the family's gonna make her come to the annual barbecue.	*conditional-NPI*
	If my little cousin plans things for Labor Day,	then the family's gonna make her come to the annual barbecue.	*conditional-bare plural*
	If my little cousin plans nothing for Labor Day,	then the family's gonna make her come to the annual barbecue.	*conditional-negative NP*
38	If my grandfather plants anything in his garden,	then my grandma is gonna go to the store for fresh vegetables.	*conditional-NPI*
	If my grandfather plants things in his garden,	then my grandma is gonna go to the store for fresh vegetables.	*conditional-bare plural*
	If my grandfather plants nothing in his garden,	then my grandma is gonna go to the store for fresh vegetables.	*conditional-negative NP*
39	If the head cook preps anything in the afternoon,	then the wait time is gonna be really long during the dinner rush.	*conditional-NPI*
	If the head cook preps things in the afternoon,	then the wait time is gonna be really long during the dinner rush.	*conditional-bare plural*
	If the head cook preps nothing in the afternoon,	then the wait time is gonna be really long during the dinner rush.	*conditional-negative NP*
40	If the lazy student reads anything over spring break,	then she's probably gonna have to catch up when she gets back.	*conditional-NPI*
	If the lazy student reads things over spring break,	then she's probably gonna have to catch up when she gets back.	*conditional-bare plural*
	If the lazy student reads nothing over spring break,	then she's probably gonna have to catch up when she gets back.	*conditional-negative NP*
41	If the young kid yells anything during the play,	then the family is gonna feel okay about staying to the end.	*conditional-NPI*
	If the young kid yells things during the play,	then the family is gonna feel okay about staying to the end.	*conditional-bare plural*
	If the young kid yells nothing during the play,	then the family is gonna feel okay about staying to the end.	*conditional-negative NP*
42	If the sixth-grader recalls anything during the test,	then his grade is gonna be really bad compared to other kids.	*conditional-NPI*
	If the sixth-grader recalls things during the test,	then his grade is gonna be really bad compared to other kids.	*conditional-bare plural*
	If the sixth-grader recalls nothing during the test,	then his grade is gonna be really bad compared to other kids.	*conditional-negative NP*
43	If the ski store sells anything to the locals,	then it's probably gonna end up closing for the off-season.	*conditional-NPI*
	If the ski store sells things to the locals,	then it's probably gonna end up closing for the off-season.	*conditional-bare plural*
	If the ski store sells nothing to the locals,	then it's probably gonna end up closing for the off-season.	*conditional-negative NP*

44	If the hotdog stand sells anything during the storm,	then the owners are gonna close it down next time it snows.	*conditional-NPI*
	If the hotdog stand sells things during the storm,	then the owners are gonna close it down next time it snows.	*conditional-bare plural*
	If the hotdog stand sells nothing during the storm,	then the owners are gonna close it down next time it snows.	*conditional-negative NP*
45	If my best friend studies anything during the review,	then she's probably gonna do really bad on the exam tomorrow.	*conditional-NPI*
	If my best friend studies things during the review,	then she's probably gonna do really bad on the exam tomorrow.	*conditional-bare plural*
	If my best friend studies nothing during the review,	then she's probably gonna do really bad on the exam tomorrow.	*conditional-negative NP*
46	If the little boy takes anything for lunch today,	then his lunchbox is gonna be totally empty when it's time to eat.	*conditional-NPI*
	If the little boy takes things for lunch today,	then his lunchbox is gonna be totally empty when it's time to eat.	*conditional-bare plural*
	If the little boy takes nothing for lunch today,	then his lunchbox is gonna be totally empty when it's time to eat.	*conditional-negative NP*
47	If the guest speaker plans anything before her speech,	then she is probably gonna sound flustered during the lecture.	*conditional-NPI*
	If the guest speaker plans things before her speech,	then she is probably gonna sound flustered during the lecture.	*conditional-bare plural*
	If the guest speaker plans nothing before her speech,	then she is probably gonna sound flustered during the lecture.	*conditional-negative NP*
48	If the famous author writes anything over the summer,	then her readers are gonna be out of books to read before long.	*conditional-NPI*
	If the famous author writes things over the summer,	then her readers are gonna be out of books to read before long.	*conditional-bare plural*
	If the famous author writes nothing over the summer,	then her readers are gonna be out of books to read before long.	*conditional-negative NP*

Filler Sentences

Table A.3 Filler sentences used in both surveys. All participants saw all filler sentences.

Filler Type	First Clause	Continuation
so	Most of the kids avoided the green vegetables at dinner,	so the kid who likes spinach is gonna get to eat the whole salad.
	The town roads were mostly made of gravel and dirt,	so the one that's actually paved is gonna get the most traffic.
	Lots of people were waiting outside the restaurant,	so the group with the reservation is gonna be happy they made one.
	The performer expects to be anxious before his show,	so he's gonna pace around backstage until it's time to go on.

	The restaurant was starting to get crowded during lunch,	so the servers are gonna be really busy with full sections.
	The neighborhood kids were scared of big dogs,	so the new family is gonna have to keep their German Shepherd inside.
	Sarah and Steven decided to clean out their t-shirt drawers,	so the shirts with holes and stains are gonna get tossed.
	A popular new movie comes out tonight at midnight,	so the movie theater is gonna be packed all day tomorrow.
	The picky eater hated tomatoes in her food,	so she's gonna refuse to eat the spaghetti her mom made.
	The plane was overbooked for the flight to New York,	so some passengers are gonna have to give up their seats today.
	The little girl's hair finally got long enough to donate,	so she's gonna have it cut for charity later this week.
	The playful kids left blocks all over the floor,	so their parents are gonna make them clean up before dinner.
	The young kid dropped his book into a puddle,	so he's probably gonna get in trouble with his teacher.
	My roommate wore her nice green dress last weekend,	so she's gonna wear a pair of blue jeans to the party today.
	The man forgot about the bananas that he had on the table,	so they're gonna be too brown for anything but banana bread.
	The young boy dropped the fishing pole into the lake,	so he's gonna try to find a way to reach it from the pier.
if-then	If the weather gets better before the weekend,	then the team is gonna be able to practice outside.
	If the rides at the amusement park need maintenance,	then they're gonna close for a few weeks over the winter.
	If the lonely dog waits for his owners by the front door,	then he's gonna know when they get home from work.
	If the angry students walk out before the end of class,	then they are gonna get in trouble with their teacher.
	If a tree limb falls on a powerline during the storm,	then a maintenance worker is gonna have to come fix it.
	If the strong wind blows the snow into the road,	then drivers are gonna need to be careful coming through.
	If the power goes out for more than a couple hours,	then the food in the fridge is gonna start going bad.
	If the props manager finds the masks for the first scene,	then the play is gonna start right on time tonight.
	If the new movie scares the family after they watch it,	then they're gonna leave the lights on when they go to bed.
	If the kitchen timer is too quiet for Mandy to hear,	then the cookies are gonna burn before she takes them out.
	If a rainstorm comes through during the wedding,	then they're gonna move the reception inside.

	If the bike tires lose too much air over the winter,	then the kids are gonna need to pump them up in the spring.
	If her new jacket comes in the mail before the weekend,	then Karen's gonna have something warm to wear on the hike.
	If the bank closes before Aaron can get off of work,	then he's gonna wait till tomorrow to deposit the check.
	If the squirrel steals food from the person on the bench,	then it's probably gonna have enough for the rest of the day.
	If the bus driver waits for the person running up to the stop,	then the bus is gonna be late when it gets to the library.
everybody/ everything	My grandma talked to everybody during her walk on Sunday,	so they're all gonna know what's going on in her life.
	The math professor gave everybody a practice exam on Tuesday,	so they're all gonna be prepared for the exam next week.
	The taxi driver told everybody how dangerous the area was,	so they're all gonna try to avoid it when they go out at night.
	Running the race made everybody tired and dehydrated,	so they're all gonna need lots of water when they finish.
	The nine-year-old invited everybody to her birthday party,	so she's gonna plan fun games for all the guests.
	My smart friend helped everybody with their homework,	so she's gonna be too tired to do a good job on her own.
	The guitar player thanked everybody who came to his gig,	so they're gonna recommend his music to their friends.
	My friend's mom knew everybody in her new book club,	so she's gonna enjoy going every week to see her friends.
	The tourists saw everything at the museum this morning,	so they're gonna spend the afternoon at the park.
	The strict professor put everything on the test,	so the students are gonna work through all their notes.
	The band played everything they knew during practice,	so they're gonna be completely ready for their next show.
	The big fish ate everything the tourists tossed in the pond,	so it's probably gonna end up getting even bigger.
	The hungry kids liked everything they saw on the table,	so it's gonna be really easy to get them to eat dinner.
	The lady bought everything she needed at the grocery store,	so she's not gonna have to go back out later.
	The clumsy kid spilled everything on his tray,	so he's gonna run and find a towel to clean it up.
	The heavy rain made everything on the patio really wet,	so the family is gonna eat dinner together inside today.
single negation	The dancer didn't break in her new shoes before the show,	so she's gonna have blisters when the night's over.
	Me and my sister didn't eat before we went to the mall,	so we're gonna get pretzels while we walk around and shop.

	The soup kitchen didn't prepare enough food for the month,	so they're gonna have another cooking day this week.
	The desk workers didn't finish enough work for the day,	so they're gonna have to work really late tonight.
	The teacher didn't open the windows in the stuffy room,	so the heat's gonna make the students sleepy during the exam.
	The leaves didn't stick to the car when they fell,	so the family is gonna be happy that it's clean.
	The hair dresser didn't clean the chair after the last client,	so her next client is gonna have to wait a little longer.
	The firefighter didn't check his gear carefully enough,	so he's gonna get in trouble with is boss for being careless.
	The singer didn't schedule the hair appointment,	so she's gonna schedule one for next week before the concert.
	The plant didn't receive enough water during the summer,	so it is gonna die before it gets to bloom in the fall.
	The gray dog didn't feel good after eating from the trash can,	so his owners are gonna wonder what's wrong with him.
	The crossing guard didn't remember her umbrella this morning,	so she's gonna get really wet while helping the kids cross the street.
	The tired doctor didn't read enough about the new heart medication,	so he's gonna get more information before recommending it.
	A lot of the fruit at the market didn't sell before the end of the day,	so the shop owners are gonna donate it to the homeless shelter.
	The new glasses didn't help the boy see better,	so he's gonna go back to the eye doctor for a new pair.
	The artist's new pencils didn't look good on the drawing paper,	so he is probably gonna buy a different kind of paper to use them on.
because	My friend Dave is dreading going into work on Monday morning,	because he's gonna have to ask his boss for a big raise.
	The pro athlete is skipping her normal morning shower,	because she's gonna go on a long run right after breakfast.
	My college roommate needs to wash her favorite shirt today,	because she's gonna wear it tonight to a friend's party.
	The curly haired girl is putting her hair up in a hat today,	because she's worried it's gonna get all wet and frizzy in the rain.
	The floor cleaner is trying to finish work an hour early tonight,	because she's gonna hang out and get food with her best friend.
	The fast food worker is worried about making french fries at work,	because he's probably gonna get splashed by the hot frying oil.
	The Canadian student is sad about going home during school break,	because he's gonna miss all of the friends he made in the US.
	The sweet grandmother is heating up milk while cleaning the kitchen,	because she's gonna make hot chocolate for her grandkids and herself.

	The carpenter was trying to find his safety glasses on his messy desk,	because he's gonna chop up some new wood that came into the shop today.
	The car mechanic did a careless job of taking off the old tire,	because he knows he's gonna have to replace the whole thing.
	The store manager showed up late to work this morning,	because she's gonna have to stay at the store really late tonight.
	The rock climber finally bought his own climbing gear at the store,	because from now on he's gonna start climbing a couple times a week.
	The math tutor took a lot of time to explain the complicated problem,	because she knows the students are gonna have it on a test tomorrow.
	The furniture movers started moving the heaviest things first,	because they know their arms are gonna be super tired later on.
	The semi-truck driver left early from the warehouse after the storm,	because he knows the driving is gonna be really slow in the snow.
	The young kids looked for fallen branches and twigs after school,	because they're gonna use them to roast marshmallows tonight.
but	My little brother's planning to get his usual chocolate ice cream,	but I'm gonna order cookies'n'cream today for a change.
	The forgetful teenager left his phone on the bus to school,	but he's gonna get it back from the bus driver later today.
	The young girl ripped her pants when she fell on the icy sidewalk,	but she's gonna go to school anyway to avoid being late.
	The little boy wants to get a new video game for his birthday,	but his parents are gonna get him something less violent instead.
	The sick student missed a test to go to the doctor's office,	but he's gonna make it up by staying late after school tomorrow.
	The shy kitten hides behind the big sofa whenever guests come over,	but she's probably gonna come out later when it's dinner time.
	The hiking group lost a few hours looking for the extra granola bars,	but they're gonna hike faster for the rest of the day to make up for it.
	The rushed teacher had to skip drinking coffee this morning to save time,	but he's definitely gonna get up early tomorrow morning for his coffee.
	The new phones cost way more money than the older phones did,	but people are still gonna buy the popular new phones when they come out.
	The artist's new paintings look really different from her old stuff,	but the paintings are gonna be really popular with a younger crowd.
	The big event at the public library starts at noon today,	but the workers are gonna have to get there at 10 am to set up.
	The new apartment building has a beautiful view of the city below it,	but the top floor renters are gonna have to climb a lot of stairs.
	The potted plants should be able to survive a little cold weather,	but the gardener is gonna bring them in before the first snow anyway.
	The potential house buyers hate the wall paper in the kitchen,	but the owners are gonna strip the walls before they move.

	The transfer students will be completely lost when they get to campus,	but a group of students is gonna help them find their way around.
	The old window slams shut whenever the renters try to open it,	but they're gonna wait to deal with it until after the cold winter is over.
mismatch/ catch trials	The office assistant went over the letter very carefully,	so there's definitely gonna be mistakes when it gets sent.
	The birthday candles typically last a very short time,	so lots of people are gonna use theirs for many years.
	The mean teacher got fired from his job at the elementary school,	so it's probably gonna be a long time before he needs another job.
	The lazy office worker takes lots of breaks during work hours,	so he's gonna get a good end of year review from his boss.
	Clients made very few appointments with the barber today,	so the barber is gonna be really busy during the day at work.
	The rushing highschooler forgot to grab her packed lunch,	so she's gonna have plenty to eat throughout the school day.
	The tall teenager feels overstuffed from eating so much at dinner,	so he's probably gonna eat a lot of dessert right after dinner.
	The designer perfume smells terrible to the girl in the store,	so she's gonna buy some for herself before she leaves the mall.
	Lots of stores in the mall closed over the past few months,	so most people are gonna keep doing all their shopping there.
	The weather channel predicts lots of rainfall for tomorrow,	so the girls are gonna plan an outdoor picnic lunch for the afternoon.
	The temperature tomorrow is supposed to stay below freezing,	so all the snow is gonna melt after the sun comes out in the morning.
	My big sister needs to have less cheese and dairy every day,	so she's probably gonna drink a lot more milk than she used to.
	The tall flowers usually break and fall whenever there's a lot of rain,	so they're gonna be standing really tall after the latest rainstorm.
	The weightlifter is used to lifting really heavy weights,	so it's gonna be hard for him to lift that really heavy barbell.
	The highschooler received a perfect score on a really hard exam,	so his parents are gonna be really angry with him when he gets home.
	The motivated student finished her writing by working really hard,	so she's gonna have to work on her writing all day tomorrow too.
